# Loss of LGR4/GPR48 causes severe neonatal salt wasting due to disrupted WNT signaling altering adrenal zonation

**DOI:** 10.1172/JCI164915

**Published:** 2023-02-15

**Authors:** Cécily Lucas, Kay-Sara Sauter, Michael Steigert, Delphine Mallet, James Wilmouth, Julie Olabe, Ingrid Plotton, Yves Morel, Daniel Aeberli, Franca Wagner, Hans Clevers, Amit V. Pandey, Pierre Val, Florence Roucher-Boulez, Christa E. Flück

**Affiliations:** 1 Laboratoire de Biochimie et Biologie Moléculaire, UM Pathologies Endocriniennes, Groupement Hospitalier Est, Hospices Civils de Lyon, Bron, France.; 2 University of Lyon, Université Claude Bernard Lyon 1, Lyon, France.; 3 Université Clermont Auvergne, CNRS, Inserm, Génétique, Reproduction et Développement, Clermont-Ferrand, France.; 4 Division of Pediatric Endocrinology, Diabetology and Metabolism, Department of Pediatrics, Inselspital, Bern University Hospital, and; 5 Department of Biomedical Research, University of Bern, Bern, Switzerland.; 6 Department of Pediatrics, Cantonal Hospital Graubuenden, Chur, Switzerland.; 7 Centre de Référence Maladies Rares du Développement Génital: du Fœtus à l’Adulte, Filière Maladies Rares Endocriniennes, Bron, France.; 8 Department of Rheumatology and Clinical Immunology/Allergology and; 9 University Institute of Diagnostic and Interventional Neuroradiology, Inselspital, Bern University Hospital, University of Bern, Bern, Switzerland.; 10 Oncode Institute, Hubrecht Institute, Royal Netherlands Academy of Arts and Sciences and University Medical Centre Utrecht, Utrecht, Netherlands.

**Keywords:** Endocrinology, Genetic diseases, Molecular biology, Mouse models

## Abstract

Disorders of isolated mineralocorticoid deficiency, which cause potentially life-threatening salt-wasting crisis early in life, have been associated with gene variants of aldosterone biosynthesis or resistance; however, in some patients no such variants are found. WNT/β-catenin signaling is crucial for differentiation and maintenance of the aldosterone-producing adrenal zona glomerulosa (zG). Herein, we describe a highly consanguineous family with multiple perinatal deaths and infants presenting at birth with failure to thrive, severe salt-wasting crises associated with isolated hypoaldosteronism, nail anomalies, short stature, and deafness. Whole exome sequencing revealed a homozygous splice variant in the R-SPONDIN receptor *LGR4* gene (c.618-1G>C) regulating WNT signaling. The resulting transcripts affected protein function and stability and resulted in loss of Wnt/β-catenin signaling in vitro. The impact of *LGR4* inactivation was analyzed by adrenal cortex–specific ablation of *Lgr4*, using *Lgr4^fl/fl^* mice mated with *Sf1:Cre* mice. Inactivation of *Lgr4* within the adrenal cortex in the mouse model caused decreased WNT signaling, aberrant zonation with deficient zG, and reduced aldosterone production. Thus, human *LGR4* mutations establish a direct link between *LGR4* inactivation and decreased canonical WNT signaling, which results in abnormal zG differentiation and endocrine function. Therefore, variants in WNT signaling and its regulators should systematically be considered in familial hyperreninemic hypoaldosteronism.

## Graphical abstract

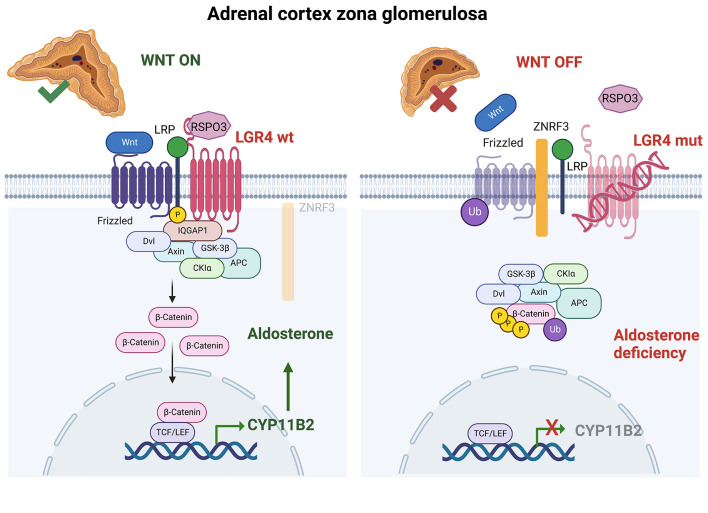

## Introduction

Disorders of isolated mineralocorticoid (MC) deficiency are potentially life-threatening (1). So far, they have been described in humans with primary defects in aldosterone biosynthesis or with MC resistance due to failure of aldosterone action. Patients mostly manifest in the neonatal period with a salt-wasting crisis, e.g., dehydration, vomiting, and failure to thrive, due to high potassium, low sodium, metabolic acidosis, and high renin. The disorder usually becomes less severe with age, as physiologic immaturity of the renal tubular system in the first year of life contributes to the impaired ability to regulate water and sodium homeostasis (2–4); beyond the neonatal period, a higher sodium intake in the diet regulated by salt appetite centrally may compensate for MC deficiency (5).

In humans, aldosterone is the principal MC produced in the zona glomerulosa (zG) of the adrenal cortex where the *CYP11B2* gene for aldosterone synthesis is expressed (6). Aldosterone synthesis, regulated by the renin-angiotensin-aldosterone feedback loop, controls salt and water homeostasis and blood pressure.

Isolated hypoaldosteronism is mostly associated with autosomal recessive variants of the *CYP11B2* gene, which catalyzes aldosterone synthesis. However, a subset of typical cases of aldosterone deficiency, grouped under familial hyperreninemic hypoaldosteronism (FHHA2), remains genetically unsolved (1). Mutations in genes that regulate aldosterone biosynthesis, downstream of renin, including genes encoding angiotensinogen (*AGT*), the angiotensin-converting enzyme (*ACE*), or the angiotensin II receptor (*AGTR1*), are associated with arterial hypotension in mice. In humans, these mutations are associated with renal tubular dysgenesis (7–10). However, they have not been linked with hypoaldosteronism. Other potential candidates comprise genes involved in the development and differentiation of the adrenal cortex.

Adrenal cortex physiology relies on functional zonation, which is essential for the production of aldosterone by the outer zG and glucocorticoids by the inner zona fasciculata (zF). The cortex undergoes constant cell renewal during postnatal life (11). This involves recruitment of subcapsular progenitor cells to zG fate and subsequent conversion to zF identity. This differentiation occurs in a centripetal manner, under the control of the WNT signaling pathway in zG and the PKA pathway in zF (12). WNT4 and R-SPONDIN3 (RSPO3) are specifically expressed in the human adrenal cortex and thus important drivers of WNT activation and zG differentiation, through stabilization of β-catenin, which stimulates expression of CYP11B2 and AGTR1 in the human adrenal cortex (13). Consequently, mouse models with *Ctnnb1*, *Wnt4*, or *Rspo3* deficiency have reduced zG differentiation (12, 14–16). Conversely, constitutive WNT pathway activation resulting from activating *CTNNB1* mutations or downregulation of negative WNT regulators is associated with the development of aldosterone-producing adenomas (17, 18). Despite the central role of canonical WNT signaling in zG differentiation, mutations in this pathway have not been associated with hypoaldosteronism in humans so far. Here, we identified a loss-of-function splice variant of the R-SPONDIN receptor–coding *LGR4* gene (c.618-1G>C) in a girl born into a highly consanguineous family with a history of multiple perinatal deaths. The proband presented with failure to thrive, severe salt-wasting crises associated with isolated hypoaldosteronism, nail anomalies, short stature, and deafness. Our in silico, in vitro, and in vivo studies established a causal link among LGR4 inactivation, decreased canonical WNT signaling, abnormal zG differentiation, and endocrine function. This suggests that anomalies in WNT signaling pathway regulators should systematically be evaluated in familial hyperreninemic hypoaldosteronism.

## Results

**Figure 1:**
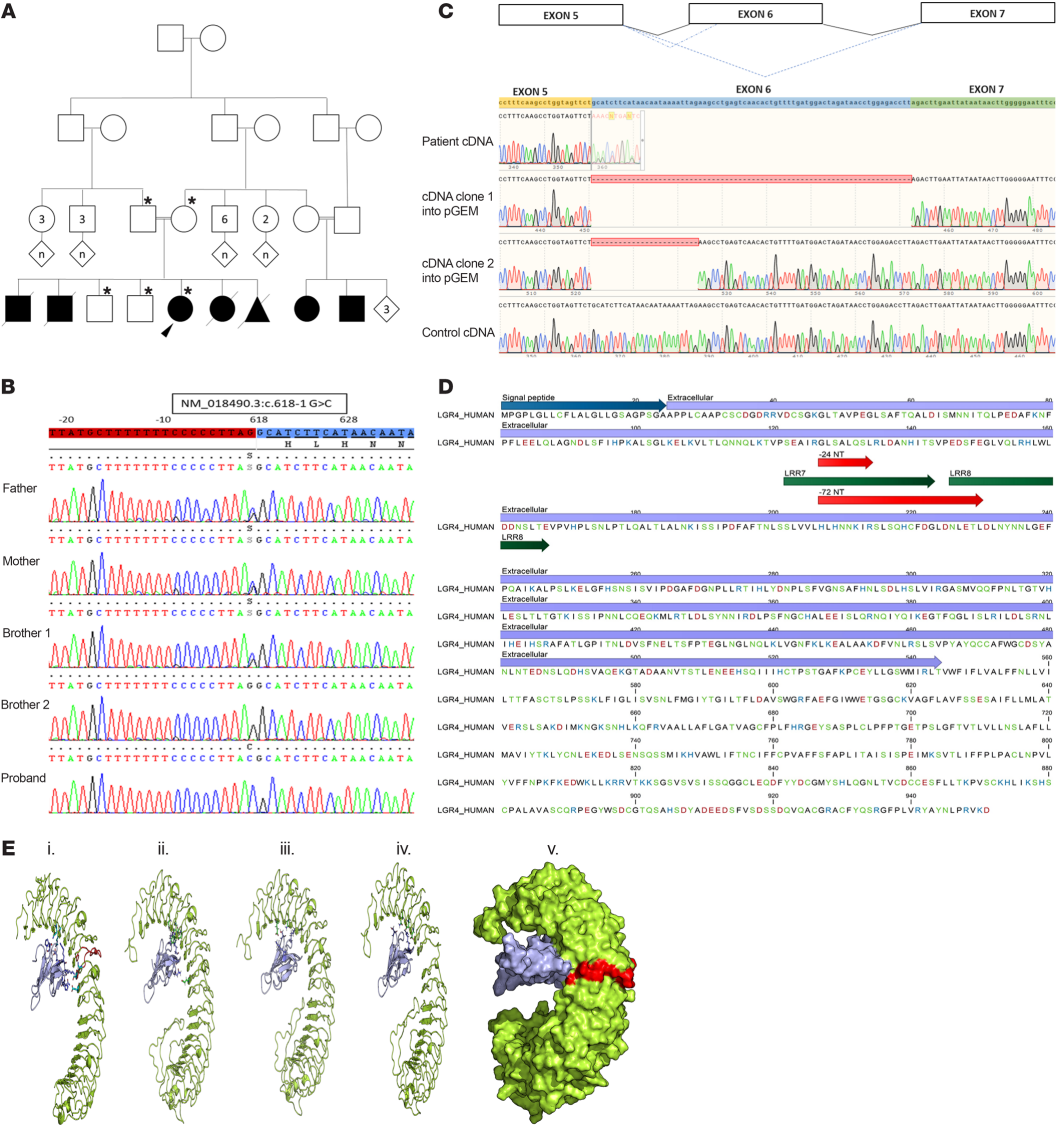
Genetic and structural characterization of a human *LGR4* mutation identified in a highly consanguineous family. (**A**) Family pedigree showing first-degree consanguinity and multiple affected individuals. Squares: male; circles: female; diamonds: unknown sex; triangle: miscarriage. Black symbols represent affected and white symbols represent unaffected individuals. Numbers in symbols represent multiple individuals. The index patient is indicated by a black arrowhead. Asterisks indicate individuals for whom DNA was sequenced. (**B**) Partial chromatograms showing the LGR4 mutation NM_018490.5:c.618-1 G>C. Intron 5 (red) and exon 6 (blue) are highlighted. The proband is homozygous, parents and 1 brother are heterozygous, and 1 brother had the WT sequence. (**C**) LGR4 mRNA analysis of proband fibroblasts. The first track represents patient mRNA after reverse transcription, indicating presence of 2 transcripts. The 2 transcripts were separated by cloning and sequenced (2 middle tracks). The bottom track shows cDNA of control fibroblasts. The scheme above shows normal splicing in dark lines and the effect of the mutation on splicing in blue dotted lines. (**D**) Amino acid sequence of extracellular domain of human LGR4 that binds to RSPOs. Amino acids found deleted in the patient are located in LRR7 (–8 AA) and LRR7/8 (–24 AA) coded by exon 6 of LGR4. (**E**) Structure of LGR4 and its interaction with RSPOs. (i) Structure of human LGR4 extracellular domain in complex with part of RSPO1 (PDB 4KT1). Amino acids coded by exon 6 are depicted in red. (ii) Human LGR4-RSPO3 complex. RSPO3 shares high structural similarity with RSPO1 and binds to LRR7 and LRR8 of LGR4. (iii and iv) Patient LGR4, missing 8 or 24 amino acids. Several critical hydrogen-bonding residues in LGR4 are missing due to mutations, causing weaker interaction and binding of RSPO3 to mutant LGR4 proteins. (v) A surface view of the LGR4-RSPO3 complex, showing close interaction points. Exon 6–encoded residues are shown in red.

In a highly consanguineous family from Syria, newborns were found to suffer from salt-wasting crises soon after birth due to isolated aldosterone deficiency. In addition, they shared a common syndromic phenotype of nail dysplasia, deafness, growth restriction, and mental disability. The index patient was referred at the age of 17 years for adrenal insufficiency, short stature, deafness, developmental delay, and dysplastic nails (Figure 1, Table 1, and Supplemental Figure 1; supplemental material available online with this article; https://doi.org/10.1172/JCI164915DS1). She was born at term, and failure to thrive and signs of an adrenal salt-wasting crisis manifested within days. Corticosteroid treatment was successfully installed without further investigations. Her parents are first-degree cousins from Syria. The presence of the same phenotype in 3 siblings who died in the neonatal period and 2 cousins (with consanguineous parents) is indicative of an autosomal recessive disorder. Laboratory workup revealed a normal cortisol response to ACTH stimulation and hyperreninemic hypoaldosteronism (Table 2). Thus, the diagnosis was revised to isolated MC deficiency; therapy was continued with fludrocortisone, while genetic workup was initiated. Additional important findings included short stature, microcephaly, structural brain anomalies, and mental disability; deafness with functional but without structural anomalies of the cochlea and hearing nerve; low bone mineral density; and small kidneys with cortical microlesions (Supplemental Figures 1–4 and Supplemental Table 1). Pubertal development was late, with menarche at 16 years.

Of note, 2 cousins with the same clinical phenotype (one female and one male) are alive and being treated with steroid replacement therapy in Syria; however, they were not available for investigations. A detailed description of the clinical findings is provided in the Supplemental Appendix.

### Identification of a human LGR4 variant.

After exclusion of *CYP11B2* mutations, filtering of the exome sequences identified a homozygous mutation in the proband *LGR4* gene: NM_018490:c.618-1G>C. Both parents and 1 brother were heterozygous for the same *LGR4* mutation but showed no overt phenotype (Figure 1B and Supplemental Table 2). The variant affects a splicing acceptor site, predicted to result in exon 6 skipping, and deletion r.618_689del or p.(His207_Leu230del). mRNA transcript analysis of the patient’s fibroblasts confirmed exon 6 skipping but also identified a second transcript with an alternative acceptor site within exon 6, leading to a shorter deletion of –24 bp, r.618_641del or p.(His207_Arg214) (Figure 1C).

### In silico analysis of LGR4 variants for prediction of pathogenicity.

Leucine-rich repeat–containing G protein–coupled receptors (LGRs) are characterized by leucine-rich repeats (LRRs) that provide the rigid structure of their large extracellular domain. LGR4 is a receptor for R-SPONDINS (RSPOs). The binding of RSPOs to LGR4 stimulates the Wnt/β-catenin signaling pathway by inhibiting the E3-ubiquitin ligases ZNRF3 and RNF43 (19). RSPOs bind to the first LRR and LRR3-LRR9 (20). The amino acid deletions caused by the loss of 8 and 24 amino acids in LGR4 are located in LRR7 and LRR8, in the extracellular domain of LGR4 (Figure 1D). A protein sequence alignment of LGR4 across species revealed that sections of LGR4 that comprise LRR7 and LRR8 were highly conserved (Supplemental Figure 5). Regions of RSPOs that interact with LGR4 were conserved among RSPOs isoforms (Supplemental Figure 6) and across species (Supplemental Figure 7). Mutations in the patient resulted in the deletion of parts of the LGR4 protein in its extracellular domain within LRR7 (for –8 AA variant) and LRR7 and LRR8 (for –24 AA variant). Structural analysis of contacts between LGR4 and RSPO1/RSPO3 showed that multiple contact points between the two proteins in the complex were located in LRR7 and LRR8 (Val204, His207, Asn226, Thr229, Tyr234, and Glu252) (Supplemental Figure 8). A loss of multiple contact points due to the deletions would result in significantly weaker interactions between LGR4 and RSPOs and affect the overall structures of complexes involving other interaction partners (ZNRF3, RNF43, UBB, UBC, etc.). In addition, the deletions were predicted to result in loss of protein stability and decreased half-life, which could alter LGR4 protein expression levels in patients. Together with weaker complex formation, lower protein levels should result in an overall loss of interaction of the –8 AA and –24 AA variants of LGR4 found in patients, with the –24 AA variant predicted to have a higher impact.

### Analysis of LGR4 protein expression in human fibroblasts.

**Figure 2:**
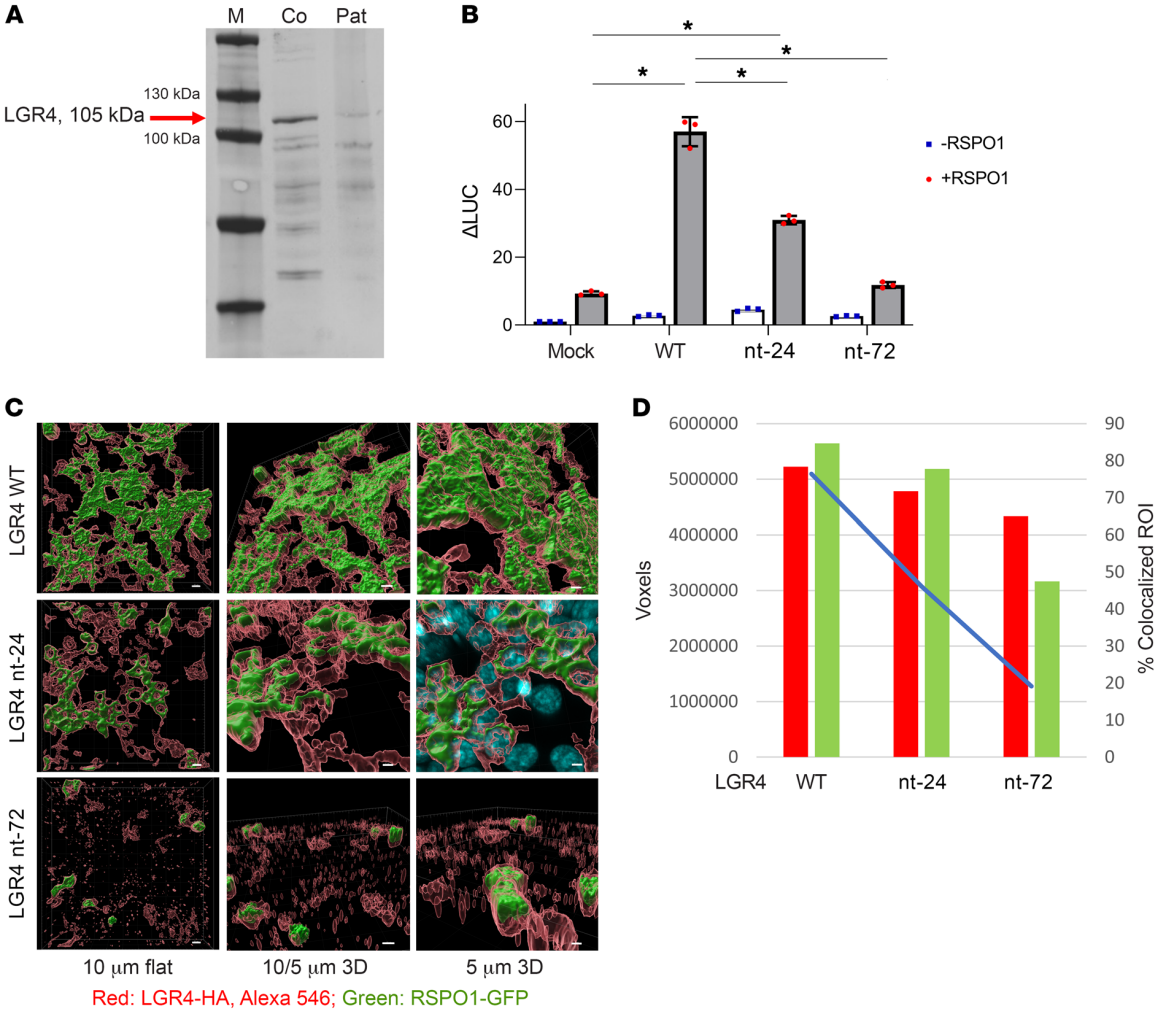
Protein expression and functional testing of Wnt/β-catenin signaling of the 2 LGR4 variants. Human fibroblasts and HEK cells were used. (**A**) Western blot analysis for LGR4 protein expression in patient and control fibroblasts. A representative blot of 3 independent experiments is shown. β-Actin was used as a loading control. The molecular weight (kDa) of a protein standard is given. M, marker; Co, control fibroblasts; Pat, patient fibroblasts. (**B**) RSPO1-activated, LGR4-mediated Wnt signaling in HEK293 cells. Cells were transfected with WT and mutant LGR4 nt-24 and nt-72 plasmids (including a mock control) and reporter vectors TOP-Flash and Renilla. Signaling was stimulated by RSPO1 and assessed by the Dual-Luciferase assay (Promega). Results are expressed as relative LUC activities (RLU). Mean ± SD of 3 independent experiments is shown. *P < 0.01, Student’s *t* test. (**C** and **D**) Interaction of RSPO1 with membrane-localized WT and variant LGR4. HEK293 cells were transfected with HA-tagged LGR4 plasmids (pcDNA3 LGR4wt, nt-24 bp, nt-72 bp) and incubated with conditioned RSPO1-GFP SN medium (previously produced in HEK cells transfected with pSpark- RSPO1-GFP). Cells were fixed with Carnoy’s solution. Staining was performed with antibody anti–HA-Tag (green) first and antibody anti-mouse Alexa Fluor 594 (red) second. Immunofluorescent microscopy was used to detect the cellular distribution of the tagged proteins as well as their colocalization (Zeiss LSM 710). Three independent experiments were analyzed. Representative images of confocal analysis at original magnification, ×40 are shown for WT and variants of LGR4. Scale bars: 5 μm (top 2 images in middle column and right); 10 μm (left and bottom image in the middle column). Quantification of colocalized LGR4 and RSPO1 was performed by Imaris (Bitplane AG).

Western blot analysis showed that patient fibroblasts expressed LGR4 protein minimally compared with controls (Figure 2A), but the expression was also low in control fibroblasts from healthy individuals. Unfortunately, biomaterial from heterozygote family members was not available.

### Functional analysis of LGR4 variants in cell models.

To assess the function of identified LGR4 variants on RSPO1 activated Wnt signaling, we used an established TOP-Flash luciferase reporter assay (21–26). When using fibroblasts, RSPO1 stimulation of endogenous LGR4/Wnt/β-catenin signaling was not strong enough for luciferase readout. Therefore, we performed studies in HEK293T cells that were transiently transfected with WT and variants of LGR4. While the basal activity of the Wnt/β-catenin signaling was low and did not differ between WT and LGR4 variants, RSPO1 activated signaling was increased 6.1-fold with WT LGR4 (*P* < 0.0001, Figure 2B). By contrast, the LGR4 variant missing 24 nucleotides from the mutant gene (referred to as nt-24 variant) increased Wnt/β-catenin signaling only 3.3-fold (*P* < 0.0001), and the LGR4 nt-72 variant completely failed to activate Wnt/β-catenin signaling (Figure 2B). Thus, compared with WT, the LGR4 nt-72 showed loss of function, while the LGR4 variant nt-24 had 54% activity. In line with these results, loss of interaction by deletion of 24 amino acids in the nt-72 variant was predicted to have a higher effect on binding as well as protein stability (Supplemental Figure 8), which explains the very low level of LGR4 protein detected in Western blots of patient fibroblasts.

### Analysis of localization of LGR4 and binding to RSPO1 in HEK293 cells.

LGR4 localizes to the cell membrane and reveals its signal-transducing functionality upon binding to RSPOs (21–27). To assess localization and RSPO1-binding characteristics of WT and mutant LGR4 proteins, we expressed HA-tagged LGR4 in HEK293 cells and studied its binding to GFP-tagged RSPO1 by confocal microscopy. As depicted in Figure 2C, LGR4 localized to the cell surface. nt-24 LGR4 was expressed at a lower level than WT LGR4, and cells carrying the nt-72 LGR4 expressed the lowest level of LGR4 (Figure 2D), in line with LGR4 protein expression in patient fibroblasts (Figure 2A). RSPO1 binding with LGR4 protein was also altered with nt-24 LGR4 compared with WT and almost absent with nt-72 (Figure 2, C and D). Altogether, these in vitro data show that the mutant LGR4 proteins identified in the proband are deficient in their ability to bind RSPOs and stimulate downstream WNT signaling.

### Lgr4 ablation results in a disrupted Wnt/β-catenin signaling pathway in mice.

**Figure 3:**
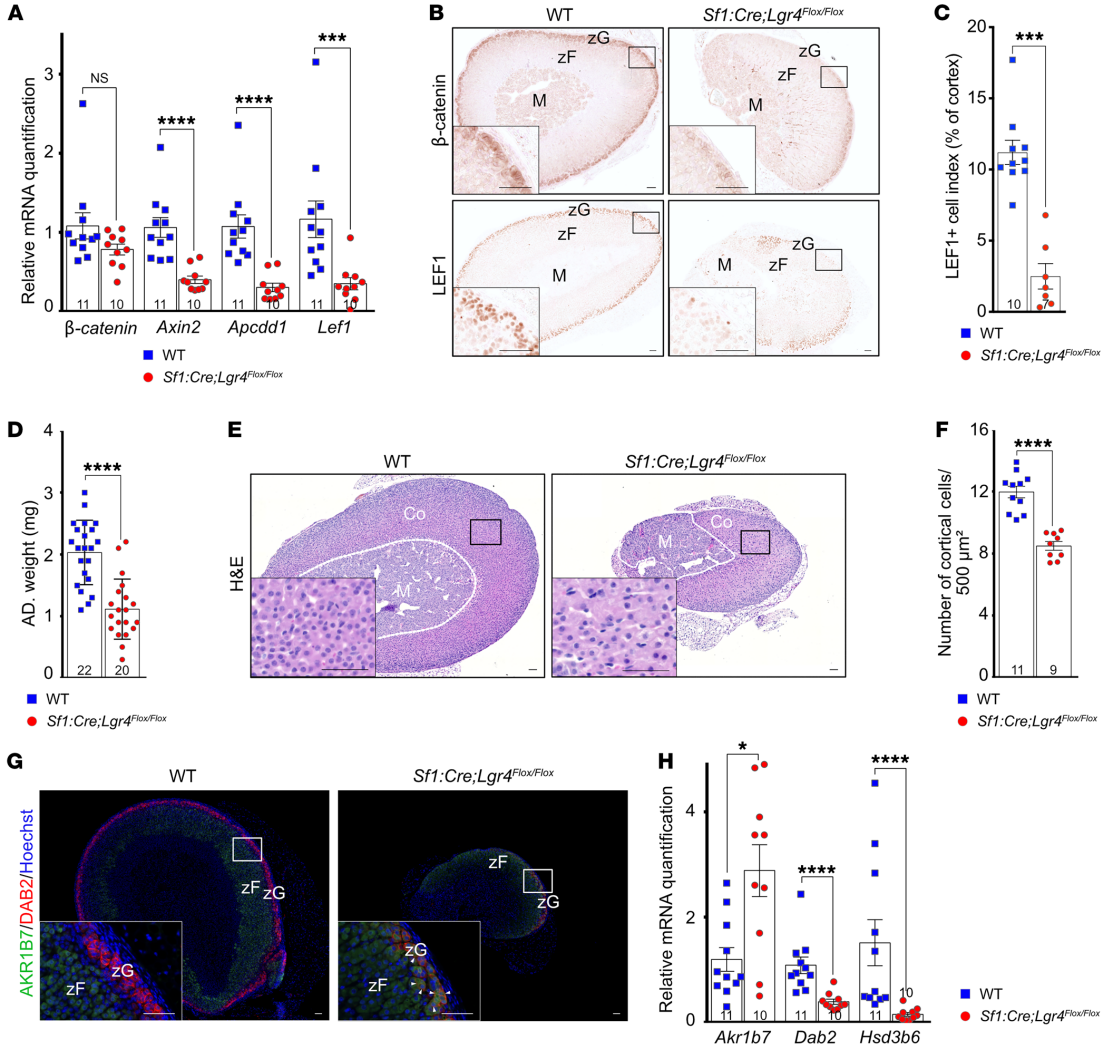
*Lgr4* ablation disrupts the Wnt/β-catenin signaling pathway, resulting in adrenal hypoplasia and aberrant zonal differentiation. (**A**) RT-qPCR analysis of mRNA encoding Wnt/β-catenin signaling pathway–associated genes. (**B**) Immunohistochemical detection of β-catenin and Lef1. (**C**) LEF1^+^ cell index, defined as the percentage of LEF1^+^ cells over the total number of cortical cells. (**D**) Adrenal weight. (**E**) H&E staining of WT and Lgr4cKO adrenals. (**F**) Number of cortical cells per 500 μm^2^ of the cortex. (**G**) Coimmunostaining for *Akr1b7* and *Dab2* in WT and Lgr4cKO adrenals. (**H**) RT-qPCR analysis of mRNA encoding zone-specific markers (*Akr1b7*, *Dab2*, and *Hsd3b6*). All analyses were conducted in 5-week-old WT and Lgr4cKO female mice. zF, zona fasciculata; zG, zona glomerulosa; M, medulla; Co, cortex. Scale bars: 50 μm. Data are shown as the mean ± SEM. Numbers of individual samples analyzed are indicated within the bars. Statistical analyses in **A**, **C**, **D**, **F**, and **H** were conducted using Mann-Whitney tests in GraphPad Prism 9. *P < 0.05, ***P < 0.001, ****P < 0.0001.

To study the role of LGR4 in adrenal function in vivo, we conditionally inactivated *Lgr4* within steroidogenic cells in the adrenal cortex of Lgr4–conditional KO mice (Lgr4cKO mice). Reverse transcription quantitative real-time PCR (RTqPCR) showed a reduction in *Lgr4* mRNA, confirming efficient deletion of the floxed allele in Lgr4cKO mice (Supplemental Figure 9A). Consistent with our in vitro data, conditional inactivation of *Lgr4* resulted in a significant decrease in expression of WNT target genes in the adrenals of Lgr4cKO mice (*Apcdd1*, *Axin2*, and *Lef1*) (Figure 3A), with decreased accumulation of both β-catenin and LEF1 proteins in the presumptive zG of mutant mice, where they normally accumulate in control mice (Figure 3, B and C).

### Lgr4 ablation causes adrenal hypoplasia and aberrant zonal differentiation.

The reduced canonical WNT signaling in Lgr4cKO mice was associated with decreased adrenal weight at 5 weeks of age (Figure 3D), massive cortical thinning, off-center localization of the adrenal medulla, steroidogenic cell cytomegaly, and a significant decrease in cortical cells numbers (Figure 3, E and F). Interestingly, adrenal cortex thinning was not associated with decreased proliferation or increased apoptosis, suggesting that it relied on altered development/maintenance of the gland (Supplemental Figure 9, B and C). Analysis of adrenal cortex differentiation by immunohistochemistry showed a marked decrease in the number of cells expressing the zG marker DAB2 and expansion of the expression domain of zF marker AKR1B7 up to the capsule, where the zG normally resides (Figure 3G). *Lgr4* deficiency also resulted in the accumulation of cells with both zG (DAB2^+^) and zF (AKR1B7^+^) identity that were not found in control mice (Figure 3G, arrowheads), demonstrating a marked impairment of adrenal cortex differentiation. Aberrant cortical differentiation was further confirmed by RTqPCR showing increased *Akr1b7* expression (zF) and decreased expression of the zG markers *Dab2* and *Hsd3b6* (Figure 3H), consistent with previous data showing decreased zG differentiation and expansion of zF in mice with decreased adrenal WNT signaling (12, 15).

### Lgr4 ablation inhibits zG zonation resulting in primary hypoaldosteronism.

**Figure 4:**
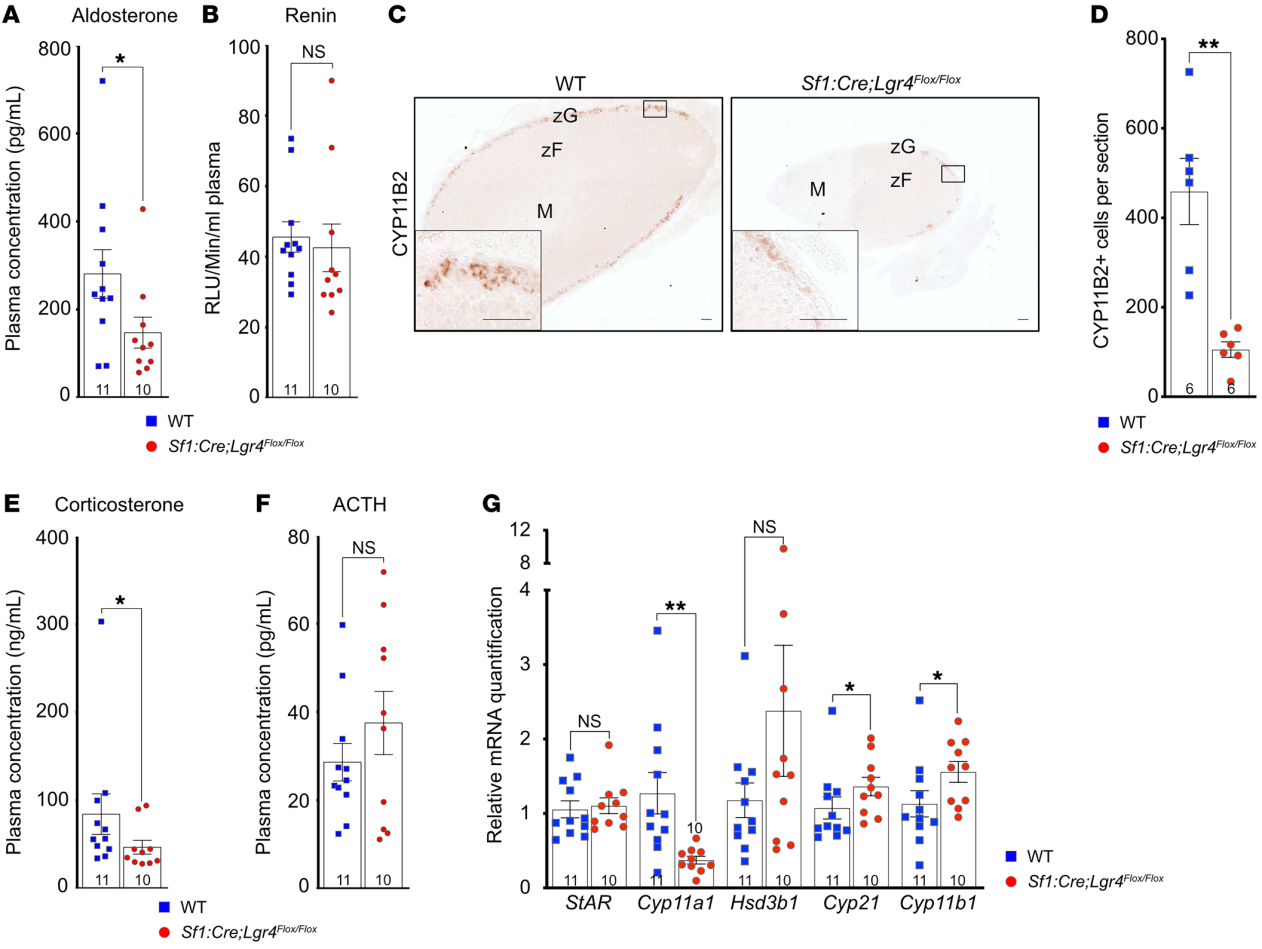
*Lgr4* ablation inhibits zona glomerulosa differentiation, resulting in primary hypoaldosteronism. (**A**) Aldosterone plasma concentration and (**B**) renin activity in 5-week-old WT and Lgr4cKO female mice. (**C**) Immunohistochemical detection of CYP11B2. Scale bar: 50 μm. zF, zona fasciculata; zG, zona glomerulosa; M, medulla. (**D**) Number of CYP11B2^+^ cells per adrenal section. (**E**) Corticosterone and (**F**) plasma ACTH concentration. (**G**) RT-qPCR analysis of mRNA encoding steroidogenesis-related genes. All analyses were conducted in 5 weeks WT and Lgr4cKO female mice. Data are shown as the mean ± SEM. Numbers of individual samples analyzed are indicated within the bars. Statistical analyses in **A**, **B**, and **D**–**G** were conducted using Mann-Whitney tests in GraphPad Prism 9. *P < 0.05, **P < 0.01.

Consistent with observations in our patient, zG differentiation anomalies in Lgr4cKO mice resulted in a significant decrease in plasma aldosterone (Figure 4A) and an increase in hematocrit, suggestive of dehydration (Supplemental Figure 9C). The observation of normal plasma renin activity (Figure 4B) suggested that hypoaldosteronism in Lgr4cKO mice was of primary adrenal origin. This was further supported by the almost complete extinction of CYP11B2 protein expression in the Lgr4cKO zG (Figure 4, C and D). Interestingly, plasma corticosterone concentration was significantly decreased (Figure 4E); this was associated with a significant decrease in *Cyp11a1* expression, which is essential for the first step of both aldosterone and corticosterone synthesis (Figure 4G). However, there was a concomitant increase in *Cyp21* and *Cyp11b1* expression, which was not associated with altered plasma ACTH concentration (Figure 4, F and G). This may reflect the aberrant expansion of zF at the expense of zG, and hence an increased ratio of zF to zG cells, rather than a direct effect of ACTH on steroidogenic gene expression.

Altogether, these data show that *Lgr4* inactivation is sufficient to significantly reduce WNT signaling in the adrenal cortex, which results in early-onset primary hypoaldosteronism.

## Discussion

Although adrenal insufficiency with MC and GC deficiencies has been reported for several complex syndromes, for which genetic variants lead to structural and/or functional defects of the adrenals and other organ systems (e.g., IMAGe, MIRAGE syndromes) (11), an inherited syndrome with isolated MC deficiency at birth, has not been described so far. In this study, we identified what we believe to be a novel syndromic form of severe neonatal salt wasting in a highly consanguineous family. In the index patient, isolated MC deficiency was diagnosed and treated successfully with MC replacement therapy, while cortisol production remained normal in the first 2 decades of life. Associated defects included nail anomalies, hearing loss, short stature, and mental disability in the index patient and both affected cousins (Figure 1A). We were aided by the consanguinity in this family, which allowed us to reveal the underlying genetic cause involving a homozygous splice site variation in the *LGR4* gene (ch11p14.1) that produces 2 shorter splice variants. LGR4, also named GPR48, is a leucine-rich repeat–containing G protein–coupled receptor, widely expressed in multiple tissues from early embryogenesis to adulthood (19, 28). LGR4 potentiates canonical WNT signaling, through inhibition of the ZNRF3- and RNF43-mediated degradation of Frizzled receptors, after binding to RSPOs (19). Consistent with this, our in vitro studies showed that the 2 aberrant *LGR4* transcripts found in the proband coded for proteins with significantly reduced activity on WNT/β-catenin signaling in vitro. We further showed that genetic inactivation of *Lgr4* within steroidogenic cells of the adrenal cortices of transgenic mice resulted in decreased canonical WNT signaling, deficient zG differentiation, and reduced aldosterone production. Even though previous reports have shown adrenal dysgenesis in patients with inactivating mutations of WNT4 in the context of SERKAL syndrome, profound developmental defects resulted in embryonic lethality, precluding evaluation of adrenal differentiation and endocrine activity (29). Therefore, to the best of our knowledge, our study is the first to demonstrate a key role of LGR4 and, more broadly, of deficient canonical WNT signaling in adrenal differentiation and zG hypofunction in patients.

Beyond primary hypoaldosteronism, the index case also presented with a spectrum of defects, including nail anomalies, hearing loss, and short stature, which were also associated with in utero death of presumably affected siblings. Our model of conditional *Lgr4* ablation within steroidogenic cells did not allow evaluation of LGR4 in these phenomena. However, studies of LGR4 variants in patients and of whole-body *Lgr4*-KO mice demonstrated the association between LGR4 alterations and fetal/perinatal death, short stature, deafness, and dysplastic nails (Supplemental Table 3). This strongly suggests that the broad defects observed in our proband are the result of the identified LGR4 mutation. So far, only individuals who carry heterozygous LGR4 variants have been described. Heterozygous human LGR4 variants are associated with low bone mineral density, electrolyte imbalance, reduced testosterone production, and increased risk of cancers of the biliary system and skin (30). More recently, 3 rare heterozygous missense variants in *LGR4* were associated with delayed puberty, resulting from alterations in the development of hypothalamic GnRH neurons (31). In line with these findings, heterozygous family members as well as our index patient had delayed pubertal onset and low bone mineral density (Supplemental Appendix Extended Case Report, Supplemental Figure 4, and Supplemental Tables 1 and 3).

Loss of LGR4 is also potentially implicated in the rare aniridia-genitourinary anomalies-mental retardation (AGR) syndrome, in which a heterozygous, contiguous gene deletion of the 11p13-14 region had been identified as comprising the *LGR4* gene. Similar to the phenotype of AGR syndrome, whole-body deletion of *Lgr4* in mice led to aniridia, polycystic kidney disease, genitourinary anomalies, and mental retardation (32). Although there were no reports of adrenal dysfunction in these cases, our results suggest that patients presenting with homozygous LGR4-associated genetic variations should be carefully evaluated for adrenal function.

Whereas Lgr4cKO mice show defects in both aldosterone and corticosterone secretion as early as 5 weeks of age, our index case presented with isolated MC deficiency in the first 2 decades of life. This phenotypic discrepancy could be accounted for by the residual activity of mutant LGR4 proteins in our patient, compared with the complete inactivation of LGR4 in the adrenal cortex of our transgenic model. However, ACTH stimulation testing of our patient at 21 years showed subclinical glucocorticoid deficiency. This suggests that the endocrine phenotype may progress toward full-fledged adrenal deficiency over time. Lineage-tracing studies in mice have shown that adrenal cortex cell renewal requires initial differentiation of progenitors into zG cells that subsequently differentiate into zF cells (33, 34). It is thus tempting to speculate that aberrant zG differentiation in our patient hampered cortical cell renewal, resulting in progressive exhaustion of the zF, associated with progressive glucocorticoid insufficiency. This warrants careful monitoring of patients initially presenting with primary hypoaldosteronism, without *CYP11B2* inactivating mutations.

In conclusion, we describe the first patients to our knowledge harboring biallelic *LGR4* variants and offer a mechanistic explanation for their life-threatening salt loss at birth, due to primary adrenal hypoaldosteronism. Our study confirms the important role of Wnt/β-catenin signaling for proper adrenal cortex zG and zF formation and function. Thus *LGR4* variants and potential variants in other genes involved in the complex network of LGR4/Wnt/β-catenin signaling should be considered in patients presenting with a salt-wasting crisis at birth, especially when manifesting with other syndromic features.

## Methods

### Genomic sequencing.

We sequenced the exome of the affected child, her parents, and her 2 unaffected brothers. Details are provided in Supplemental Appendix. Next-generation sequencing data have been deposited in the European Genome-phenome Archive (EGA; https://ega-archive.org/), which is hosted by the European Bioinformatics Institute and the Centre for Genomic Regulation, under accession EGAS00001006808.

### Bioinformatic and laboratory studies.

Primary fibroblasts of skin biopsies from the proband and healthy controls permitted *LGR4* transcript analysis and studies of protein expression. The putative impact of the specific LGR4 variants was analyzed in silico using the 3D structure of the human LGR4 extracellular domain in complex with a part of R-SPONDIN (PDB 4KT1). The function of identified *LGR4* variants was analyzed in vitro, using the TOP-Flash WNT signaling luciferase reporter assay (21–26) in the presence or absence of RSPO1. Localization of mutant LGR4 and interaction with RSPO1 was investigated by confocal microscopy in HEK293 cells expressing HA-tagged LGR4 and GFP-tagged RSPO1. The impact of LGR4 inactivation in vivo was analyzed by adrenal cortex specific ablation of *Lgr4*, using *Lgr4^fl/fl^* mice mated with *Sf1:Cre* mice. Full experimental details are provided in the Supplemental Appendix and Supplemental Methods.

### Statistics.

Results are shown as the mean ± SEM. The D’agostino and Pearson normality test demonstrated the absence of normality of the data. Therefore, statistical analyses between 2 or several groups were performed using Mann-Whitney or Kruskal-Wallis, respectively, using GraphPad Prism 9. Student’s 2-tailed *t* test was used for the analysis of cell experiments. A *P* value below 0.05 was considered statistically significant.

### Study approval.

Written informed consent was obtained from all study participants. Written informed consent was also received for the use of photographs of patient. Studies in humans or on human material were conducted in accordance with and with the approval of Swissethics, Switzerland (KEK Bern ID 04/07). Animal experiments were approved by the CEMÉA Auvergne ethics committee, Clermont-Ferrand, France, and the French Ministry of Agriculture’s committee on animal experimentation (APAFIS 39127).

## Author contributions

CL performed experiments on mouse biomaterials, analyzed data, created figures, and contributed to manuscript writing. KSS performed experiments on human biomaterials, analyzed data, created figures, and contributed to manuscript writing. MS performed the clinical workup of patients and contributed to manuscript writing and review. DM performed genetic test and analysis and contributed to manuscript writing. AVP provided bioinformatic analyses and predictions, created figures, and contributed to manuscript writing and review. JW performed experiments on mouse biomaterials. JO performed experiments on mouse biomaterials. IP performed hormonal tests. YM performed genetic testing and analysis. DA performed the clinical workup of patients (bone phenotype) and reviewed the manuscript. FW performed the clinical workup of patients (brain, cochlea phenotype) and reviewed the manuscript. HC provided the mouse model. PV was a study PI, provided overall design, provided organization, analyzed and interpreted data, and wrote the manuscript. FRB was a study PI, provided overall design, provided organization, analyzed and interpreted data, and wrote the manuscript. CEF was a study PI, provided overall design, provided organization, analyzed and interpreted data, and wrote the manuscript. CL, KSS, and MS share the first author position in the given sequence based on the evaluation of their specific contribution to the project, with regard to work load and significance.

## Supplementary Material

Supplemental data

## Figures and Tables

**Table 2:**
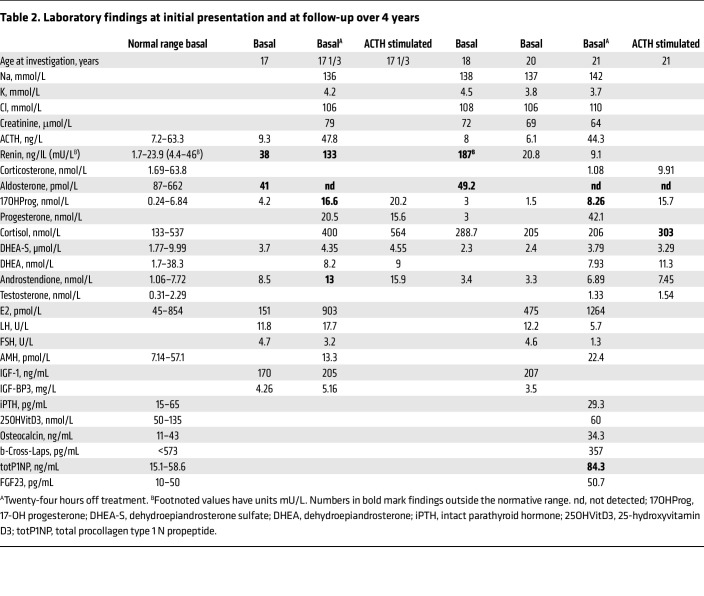
Laboratory findings at initial presentation and at follow-up over 4 years

**Table 1:**
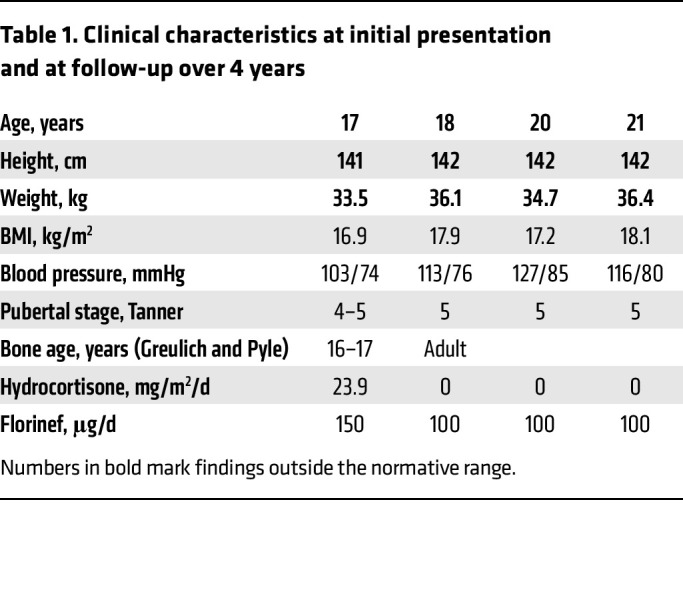
Clinical characteristics at initial presentation and at follow-up over 4 years

## Version 1 12/20/2022

In-Press Preview

## Version 2 02/15/2023

Electronic publication

## Version 3 02/20/2023

Error corrected in authorship note. Pierre Val, Florence Roucher-Boulez, and Christa E. Flück are co–last authors and contributed equally to this work.
