# Supplementary material for: Loss of LGR4/GPR48 causes severe neonatal salt wasting due to disrupted WNT signaling altering adrenal zonation
Source: J Clin Invest. 2023 Feb 15;133(4):e164915. doi: 10.1172/JCI164915 (PMC9927937; doi:10.1172/JCI164915)
Supplement: Supplemental data [file jci-133-164915-s007.pdf]

Supplementary Appendix

Loss of human LGR4/GPR48 causes a novel syndromic form of severe neonatal salt-wasting due to disrupted WNT signaling altering adrenal zonation

Table of content Page

Extended Case Report 2

Supplementary Material and Methods 4

Supplementary Figures:

Figure S1. Photo phenotype at age 21 years 11

Figure S2. Ultrasound of kidneys at age 21 years 12

Figure S3. Summary of MR imaging findings of the brain and inner ear structures 13

Figure S4. Bone morphometric findings in the index patient and family carriers 14

Figure S5. Conservation of amino acids forming LRR7 and LRR8 of LGR4 15

Figure S6. Conservation of R-SPODIN regions interacting with LGR4 across R-SPONDIN isoforms 16

Figure S7. Conservation of R-SPODIN regions interacting with LGR4 across species 17

Figure S8. Specific impact of identified LGR4 deletion variants on interaction with R-SPONDIN 18

Figure S9. Lgr4 ablation in mice results in disrupted Wnt/β-catenin signaling pathway, and causes adrenal hypoplasia and aberrant zonal differentiation 20

Supplementary Tables:

Table S1. Bone morphometrics in individuals carrying homozygous (index) and heterozygous *LGR4* variants 21

Table S2. List of alternative variants segregating with the clinical phenotype and an autosomal recessive mode of inheritance 23

Table S3. Phenotype of *LGR4* variants in humans and KO mice 24

Table S4. List of antibodies used for immunohistochemistry 27

Table S5. List of primers used for quantitative real-time PCR analysis 27

Extended Case Report

The index patient was born after an uneventful pregnancy at term, to highly consanguineous parents in Syria during war time. Birth weight was normal (3400g). At birth, she presented nail dysplasia. In the first days of life, she rapidly lost weight and became very weak due to failure to thrive and dehydration. As two older siblings died in the same hospital soon after birth with similar problems, she was quickly transferred to a bigger hospital for further diagnostic tests (results not available). Diagnosis of primary adrenal insufficiency (PAI) was made and treatment with mineralocorticoids (MC) and glucocorticoids (GC) (unknown preparations and doses) was started successfully. After 40 days in the hospital, she was discharged. Steroid treatment was mostly available to her during infancy and childhood, although difficult to import into Syria, and mostly supplied as different GC preparations, administered at different doses. At around 2 months of age, she was also diagnosed with deafness. In addition, parents describe that she was growing very slowly and showed short stature compared. Finger and toes nails remained dysplastic. Her psychomotor and intellectual development was also delayed. However, this may have been confounded by her hearing impairment and the fact that she never went to kindergarten or any kind of school for hearing impaired/deaf children. She showed first signs of puberty at around the age of 13 years and menarche at age 16 years with normal menstrual cycles thereafter. At age 17, the family moved to Switzerland as refugees from Syria. Upon entry, she was seen and treated for a suspected adrenal crisis in an emergency room, before a first pediatric assessment was performed at a local hospital (first visit in Table 1). She was then referred to our University hospital for further diagnostic work-up of primary adrenal insufficiency of unknown etiology.

At first presentation, we saw a 17 3/12 years-old woman with deafness (**Suppl Figure S1**). Height was 141 cm (- 3.1 SD), weight 33.5 kg, BMI 16.8 kg/m^2. Head circumference 49 cm (-3.5 SD); blood pressure 103/74 mmHg, HR 88/min. Physical exam revealed low subcutaneous fat, nail aplasia (fingers and toes), typical external female genitalia, breast Tanner B4, pubic hair P4-5. At presentation, she was on treatment with 27.5mg hydrocortisone/d, 150µg/d fludrocortisone and 2000IE vitamin D.

Family history revealed that parents were first cousins (**Figure 1**). Father's height was 170 cm; he reported normal puberty but was diagnosed with metabolic syndrome in middle age (diabetes type 2, obesity, high blood pressure). The mother's height was 148.5 cm; she had menarche at age 16 years with regular menstrual cycles and fertility thereafter; menopause was reported at age 50 years. The first two older brothers died after uneventful pregnancy soon after birth at day 8 and 20, respectively. They were both noted to have nail dysplasia. The next two older brothers were born without similar anomalies and developed normally. At

age 22 and 20 years they are healthy (height 170 cm and 171 cm, respectively); no pubertal delay was noted. Another sister was born two years after the index patient but died at 15 days of life, revealing the same phenotype. She was not offered treatment. In addition, the extended family comprises two affected cousins (one female and one male). They are the progeny of another first cousins' marriage and show the same phenotype as our index patient (**Figure 1**). They are alive under steroid replacement therapy but live in Syria, where they are unfortunately unavailable for further studies.

In the index patient, laboratory work-up 24 hours after removal of replacement steroids, revealed normal electrolytes, highly elevated renin and unmeasurable aldosterone, indicating MC deficiency (**Table 1**). In contrast, baseline ACTH and cortisol were normal, and cortisol increased normally with ACTH stimulation. All other measured GC and androgen metabolites were normal.

Further investigations included brainstem electric response audiometry (BERA) and otoacoustic emission testing, which showed highly pathologic results, consistent with deafness. Echocardiography was normal. Ultrasound of kidneys showed a lack of differentiation between cortex and mark as well as microcalcifications in the cortex; adrenals were not visualized, while inner female sex organs and other abdominal organs were all normal.

Following these investigations, the diagnosis of PAI was revised to isolated mineralocorticoid deficiency (MC), and consequently hydrocortisone therapy was tapered off successfully. Under isolated mineralocorticoid therapy the patient has been doing fine over the following years. (**Table 1**).

At the most recent follow-up at age 21 years revealed mostly unchanged clinical findings with the exception of insufficient cortisol response to ACTH stimulation testing (**Table 1**). Menstrual cycles were still regular. Kidneys were small by ultrasound and showed multiple cortical microlesions either equivalent to microcalcifications or microcysts (**Suppl Figure S2**). MR imaging (1.5T Siemens Avanto) with acquisition of native and post contrast high resolutions sequences of the brain, inner ear and orbits revealed unspecific anomalies of the brain consisting of incomplete myelination and cerebral maturation, but normal structure of the inner ear and the cochlea nerve on both sides (**Suppl Figure S3**). Bone morphometric investigations showed low bone mineral density in the index patient as well as in the heterozygote brother and mother (**Suppl Figure S4 and Suppl Table S1**) [1, 2].

Of special note, the patient and all relatives gave written informed consent that the photographs of the index patient and the clinical data given in Suppl Figures S1-S4 and Suppl Table S1 are shown in this report. This record of informed consent will been retained.

Supplementary Material and Methods

Genomic sequencing

Studies in humans or on human material were conducted in accordance with swissethics (KEK Bern ID 04/07). Written Informed consent was obtained from all study subjects. Pathogenic mutations in the *CYP11B2* gene were ruled out for the proband by using a classical Sanger method on DNA extracted from whole blood. Genomic DNAs were then tested by whole exome sequencing for the proband, her parents and her two brothers. Libraries were built using SeqCap EZ MedExome kit (Roche NimbleGen Madison, Wisconsin, USA) and paired-end sequencing 2x150bp was performed on a Nextseq 500 (Illumina, San Diego, CA, USA). For each sample, 1 µg of high-quality genomic DNA was fragmented with a Covaris M220 instrument (Covaris, Woburn, MA, USA). Library preparation was performed with the Kapa Library Preparation Kit for Illumina platforms (Kapa Biosystems, London, UK). The manufacturer's DNA sample preparation protocol for Roche NimbleGen SeqCap EZ Library (Roche, SeqCap-EZ_UGuide_v5p0) was followed, using single index adapters (SeqCap Adapter Kit A et B) allowing the study of 24 samples simultaneously. Validation of enrichment and quantification of enriched target DNA were performed both on the Caliper LabChip GX using the High Sensitivity assay Kit (Caliper LifeSciences Waltham, Massachusetts, USA) and the Qubit Fluorometric Quantitation DSDNA HS Assay kit (ThermoFisher Scientific, Illkirch, France) according to the manufacturers' instructions. The libraries were paired-end sequenced (2x150 pb) on a NextSeq500 sequencer (Illumina, San Diego, CA, USA).

The pipeline followed the GATK Best Practices recommendations provided by the Broad Institute (https://gatk.broadinstitute.org/hc/en-us/sections/360007226651-Best-Practices-Workflows). For each sample, read pairs were first trimmed using trimmomatic v 0.33 [3] . Then, reads were aligned against the hg19 version of the human genome using BWA-MEM v 0.7.12 [4] , producing for each sample a BAM file which was indexed and sorted with samtools v 1.3.1 [5] . Mapped reads then underwent several treatments: (i) duplicate marking was performed by PicardTools MarkDuplicates v 1.138 (Broad Institute at http://broadinstitute.github.io/picard; accessed 19 Sept 2018); (ii) indel realignment and (iii) nucleotide recalibration were done using GATK IndelRealigner and GATK BaseRecalibrator, respectively [6]. FASTQ and BAM metrics were collected using FastQC (Andrews, 2010; http://www.bioinformatics.babraham.ac.uk/projects/fastqc) and PicardTools, respectively (Picard Toolkit, 2018; Broad Institute, GitHub Repository;

http://broadinstitute.github.io/picard/). Variant calling was performed using GATK HaplotypeCaller [6], producing a genomic VCF for each sample. Genotyping was performed using GATK GenotypeGVCFs [6], merging all the samples in a unique VCF. Variants' normalization and annotation (gnomad, hgmd, clinvar, omim, dbNSFP were handled by GATK LeftAlignAndTrimVariants [6], and the snpEff/SnpSift toolbox, respectively [7] . VCF metrics were collected using snpEff/SnpSift (Cingolani, 2012). DeCovA, an in-house script, was used for copy number variant detection. GenoFilter (an in-house script) was used to filter the identified variants according to the hypothesized possible inheritance patterns (autosomal recessive in a consanguineous family). We filtered for variants that had a depth lower than 5X and >0.01 in gnomAD control databases. This left 187 variants. Only variants annotated as missense variant, splicing region variant and nonsense variant were kept (17 variants) and annotated with a moderate or high impact, leaving 9 variants (**Suppl Table S2**). Variants were classified according to the ACMG criteria and expression of the genes in the adrenal was searched in GTEx portal. The variant annotated with the highest impact was in the *LGR4* gene: NM_018490.3: c.618-1G>C. Sanger sequencing confirmation was performed for the variant and to confirm segregation of the variation. Both parents and one brother were heterozygous, the proband was homozygous and one brother had no variation (**Figure 1**).

Analysis of messenger RNA expression

For the index patient, we also obtained skin biopsy material to culture fibroblasts. For transcript analysis, RNA was isolated from patient and control fibroblasts (Qiagen RNeasy mini Kit, #74106, Hombrechtikon, Switzerland), reverse transcribed (ImProm-II™ Reverse Transcriptase, #A3801, Promega, Dübendorf, Switzerland), cloned (pGEM®-T Easy, #A1360, Promega) and then characterized by direct Sanger sequencing (Microsynth, Balgach, Switzerland).

Analysis of LGR4 protein expression in patient and control fibroblasts

Western blots were performed with 30 µg of total protein extracted from fibroblasts. Proteins were separated on 8-16% precast gel from GenScript (Leiden, Netherlands) and blotted on Immobilon FL transfer membrane (Millipore IPFL00010, Merck KGaA, Darmstadt, Germany). Standard western blot was performed with rabbit anti-human LGR4 antibodies 1:500 (Sigma SAB2701954, Merck) using Licor IRDye 680RD 926-68071 1:500 as a secondary antibody (Lincoln, Nebraska USA). For endogenous control, we used anti-beta-Actin 1:2000 (Sigma A1978) and secondary antibody Licor IRDye 800CW 926-32210 1:500. Protein bands were visualized with the Licor Odyssey SA apparatus.

In silico analysis of LGR4 transcript variants

For the LGR4 and RSPO structure analysis, three-dimensional crystal structure of human LGR4 extracellular domain in complex with a part of RSPO1 from the protein structure database (PDB # 4KT1) was used. First, the sequences of different LGR isoforms as well as analogs of LGR4 from different species were downloaded from the NCBI protein database to compare position and sequence conservation of mutated arginine. We used human (H. sapiens NCBI # NP_060960), cattle (B. taurus, NCBI #NP_001192440), mouse (M.musculus NCBI # NP_766259) and rat (R.norvegicus NCBI # NP_775450), among a range of other species for the cross-species conservation analysis. Sequence alignments were performed with ClustalW [8]. After generating a structural alignment, using multiple iterations with PhiBLAST [9], model building was performed using AMBER [10]. Sidechains of modeled residues missing in temple structures were optimized by the screening of rotamer libraries [11] and molecular dynamics (MD) simulations using YASARA [12]. The protein structures were then subjected to 500 steps of steepest descent and simulated annealing minimizations using AMBER force filed [13] and TIP3P water model [14], followed by 1000 ps of explicit solvent MD simulations at 310 K. Structural analysis was performed after the MD simulation systems were stable. All illustrations were prepared with PyMOL (www.pymol.org) and rendered as ray-traced images using PovRay (www.povray.org). Models of two different mutants with 8 and 24 amino acid deletions were built from scratch to account for proper folding with modified amino acid sequence. Separate models of three different versions of LGR4 (WT, -8AA and -24AA) were created for all structural analysis to study the interaction of LGR4 with RSPO proteins and the impact of deletion mutations in LGR4 on the loss of interaction with RSPO proteins. Sequences of RSPO isoforms in human as well as different RSPO analogs in other species were analyzed for conservation of sequences at the contact points of LGR4 and RSPO proteins in the complex structure. Models of RSPO3 were generated using AlphafoldAI [15] as well as RoseTTa fold [16] and then combined into a single model using threading with the YASARA [17]. Docking of RSPO3 on LGR4 extracellular domain was performed with AUTODOCK-Vina [18] and contact analysis between LGR4 and RSPO1/RSPO3 was performed with LigPlot [19].

Functional studies of LGR4 variants on Wnt signaling in patient and control fibroblasts, and HEK293T cells

Fibroblasts were seeded in 12 well plates at a density of 400 000/well. Cells were transfected with TOP Flash or FOP Flash plasmids (400 ng/well) and pRL-TK (10 ng/well) using Lipofectamine 3000 reagent according to the manufacturer’s instruction (Thermo Fisher Scientific (Schweiz) AG, Basel, Switzerland). The M50 Super 8x TOPFlash vectors were a gift from Randall Moon who developed this beta-catenin-mediated transcriptional activation

assay (Addgene plasmid # 12456; http://n2t.net/addgene:12456; RRID:Addgene_12456). After 24h, 100ng RSPO1/ml (human SRP3292, Sigma) was added for 21h. Firefly and Renilla luciferase signals were measured with the Dual-Luciferase Reporter Assay Kit (Promega) according to the protocol provided by the manufacturer.

Similarly, HEK 293T cells (CRL-3216™, American Tissue Culture Collection; Manassas, VA, USA) were seeded in 12-well plates at a density of 400 000/well. Cells were transfected with pcDNA3-LGR4 wt, pcDNA3-LGR4 mt-24bp or pcDNA3-LGR4 mt -72bp plasmids or pcDNA3 control plasmid (1ug/well), along with TOP Flash or FOP Flash plasmid (1ug/well) and pRL-TK (20 ng/well) using Lipofectamine 3000 reagent according to the manufacturer's instruction. After 48h, 100ng RSPO1/ml was added for 6h. Luciferase and Renilla signals were measured with the Dual-Luciferase Reporter Assay Kit (Promega) according to the protocol provided by the manufacturer. At least three independent experiments were performed in duplicates and data summarized.

Solution-based and cell-based LGR4 – RSPO1 localization and binding studies

HEK293T cells were transiently transfected (Lipofectamine 3000) with pcDNA3-LGR4 wt, pcDNA3-LGR4 mt-24bp or pcDNA3-LGR4 mt -72bp plasmids comprising C-terminal HA-tagged LGR4 for cell-based binding assays. In parallel, RSPO1-GFP was produced in HEK293T cells by transient transfection using a plasmid containing human RSPO1cDNA in pCMV3-C-GFPSpark (SinoBiological Co., Ltd, Beijing, China). Centrifuged cell supernatant was harvested after 48 hours. Then, LGR4 transfected cells were incubated with RSPO1-GFP conditioned medium for 1 h at 37°C. Cells were washed with PBS, fixed with Carnoy's solution, stained with anti-HA-Tag antibodies (anti-HA-Tag A00187 GenScript; anti-mouse Alexa 546 Thermofisher), and analyzed by fluorescent confocal microscopy. Quantitative analysis of protein localization and interaction was performed using Imaris (Bitplane AG, Zurich, Switzerland). Confirmatory analysis was performed with CellProfiler (Broad Institute). Three independent experiments were performed and data summarized.

Mouse model and study methods

The *Lgr4* knock-out mice were generated by mating *Lgr4$^{Flox/Flox}$* mice generated by Hans Clevers group [20] with *Sf1:Cre* mice generated by Bingham and colleagues [21]. Animals were maintained in a 12 light / 12h dark cycle. Data were generated from 5 weeks old female mice, maintained on mixed background (mostly composed of C57BL6/J). To ensure consistency, control mice were littermates of knockout mice. At 5 weeks of age, 10 *Lgr4* KO mice and 11 of their control littermates were euthanized by decapitation and blood was collected in vacuum blood collection tubes (VF-053STK, Terumo). For each mouse, adrenals

were either frozen in liquid nitrogen for molecular analysis or fixed in 4% PFA for immunohistochemical analyses.

Immunohistochemistry of mouse tissues

Immunohistochemistry was performed on tissues embedded in paraffin, after unmasking by boiling for 20 min in sodium citrate 10 mM, Tween 0.05% or Tris 10mM, EDTA 1mM, pH 9.0 depending on the primary antibody and/or combinations of antibodies to be used. For CYP11B2, unmasking with Tris-EDTA was followed by 5 min incubation in 10% SDS. After blocking for 1h, slides were incubated overnight at room temperature, with primary antibodies at the indicated concentrations (**Supplementary Table S4**). Primary antibodies were detected with appropriate polymers (ImmPress Polymer Detection Kit, Vector Laboratories). Polymer coupled HRP activity was then detected with either Vectastain ABC (PKD4000, Vector Laboratories) for brightfield images or TSA Alexa coupled fluorochromes for fluorescence (Invitrogen). Images were acquired with a Zeiss Axioscan Z1 slide scanner. Cell counting and adrenal surface measurements were done using QuPath software (https://qupath.github.io) [22] . CZI images generated by the slide scanner were extracted with identical setting across genotypes, using Zen software (Carl Zeiss AG). Extracted TIFFs were then minimally processed for global level and white balance using Affinity Photo®. Image settings and processing were identical across genotypes.

Total RNA extraction, cDNA synthesis, and qRT-PCR

Total mRNAs were extracted from mouse adrenals using RNeasy Micro Kit (Qiagen) according to the manufacturer's instructions. Five hundred nanograms of mRNAs were reverse transcribed for 1 hour at 37°C with 5 pmol of random hexamers primers, 200 units reverse transcriptase (MDMLV RT, M1701, Promega), 2 mM dNTPs and 20 units RNAsin (N2615, Promega). q-PCR was performed using two microliters of a one-tenth dilution of cDNA using the SYBR qPCR Premix Ex Taq II Tli RNase H+ (TAKRR820W, #Takara) and 200nM of specific primers (**Supplementary Table S5**). For each experiment and primer pair, the efficiency of PCR reactions was evaluated by amplification of serial dilutions of a mix of cDNAs. Relative gene expression was calculated using the ΔΔCt method with normalization to expression of *tbp*.

Hormone measurements in mice

Plasma aldosterone and corticosterone concentrations of mice were determined using commercially available ELISA kits (CAN-ALD-500, DBC; AR E-8100, Eurobio, respectively), following the manufacturer's instructions. Renin activity rate in plasma was determined using

a Fluorimetric Sensolyte 520 Mouse Renin Assay Kit (AnaSpec Inc). ACTH, LH and FSH concentrations were determined using a multiplex assay (MPTMAGD-49K, Merck Millipore).

Statistical analysis

Results are presented as means +/- SEM. The D'agostino and Pearson normality test demonstrated the absence of normality of the data. Therefore, statistical analyses between two or several groups were performed using Mann-Whitney or Kruskal-Wallis, respectively, using GraphPad Prism 7. A P value below 0.05 was considered statistically significant. *$P<0.05$; **$P\leq0.01$; ***$P\leq0.001$.

1. Shuhart CR, Yeap SS, Anderson PA, Jankowski LG, Lewiecki EM, Morse LR, et al. Executive Summary of the 2019 ISCD Position Development Conference on Monitoring Treatment, DXA Cross-calibration and Least Significant Change, Spinal Cord Injury, Peri-prosthetic and Orthopedic Bone Health, Transgender Medicine, and Pediatrics. J Clin Densitom. 2019; **22**(4): 453-71.
2. Rauch F, Schoenau E. Peripheral quantitative computed tomography of the proximal radius in young subjects--new reference data and interpretation of results. J Musculoskelet Neuronal Interact. 2008; **8**(3): 217-26.
3. Bolger AM, Lohse M, Usadel B. Trimmomatic: a flexible trimmer for Illumina sequence data. Bioinformatics. 2014; **30**(15): 2114-20.
4. Li H, Durbin R. Fast and accurate long-read alignment with Burrows-Wheeler transform. Bioinformatics. 2010; **26**(5): 589-95.
5. Li H, Handsaker B, Wysoker A, Fennell T, Ruan J, Homer N, et al. The Sequence Alignment/Map format and SAMtools. Bioinformatics. 2009; **25**(16): 2078-9.
6. DePristo MA, Banks E, Poplin R, Garimella KV, Maguire JR, Hartl C, et al. A framework for variation discovery and genotyping using next-generation DNA sequencing data. Nature genetics. 2011; **43**(5): 491-8.
7. Cingolani P, Platts A, Wang le L, Coon M, Nguyen T, Wang L, et al. A program for annotating and predicting the effects of single nucleotide polymorphisms, SnpEff: SNPs in the genome of Drosophila melanogaster strain w1118; iso-2; iso-3. Fly (Austin). 2012; **6**(2): 80-92.
8. Madeira F, Park YM, Lee J, Buso N, Gur T, Madhusoodanan N, et al. The EMBL-EBI search and sequence analysis tools APIs in 2019. Nucleic Acids Research. 2019; **47**(W1): W636-W41.
9. Altschul S. Gapped BLAST and PSI-BLAST: a new generation of protein database search programs. Nucleic Acids Research. 1997; **25**: 3389–402.
10. Case DA, Cheatham III TE, Darden T, Gohlke H, Luo R, Merz Jr. KM, et al. The Amber biomolecular simulation programs. Journal of Computational Chemistry. 2005; **26**(16): 1668-88.
11. Canutescu AA, Shelenkov AA, Dunbrack RL, Jr. A graph-theory algorithm for rapid protein side-chain prediction. Protein Sci. 2003; **12**(9): 2001-14.
12. Krieger E, Vriend G. New ways to boost molecular dynamics simulations. J Comput Chem. 2015; **36**(13): 996-1007.
13. Debiec KT, Cerutti DS, Baker LR, Gronenborn AM, Case DA, Chong LT. Further along the Road Less Traveled: AMBER ff15ipq, an Original Protein Force Field Built on a Self-Consistent Physical Model. Journal of Chemical Theory and Computation. 2016; **12**(8): 3926-47.
14. Duan Y, Wu C, Chowdhury S, Lee MC, Xiong G, Zhang W, et al. A point-charge force field for molecular mechanics simulations of proteins based on condensed-phase quantum mechanical calculations. J Comput Chem. 2003; **24**(16): 1999-2012.

15. Jumper J, Evans R, Pritzel A, Green T, Figurnov M, Ronneberger O, et al. Highly accurate protein structure prediction with AlphaFold. Nature. 2021.
16. Baek M, DiMaio F, Anishchenko I, Dauparas J, Ovchinnikov S, Lee GR, et al. Accurate prediction of protein structures and interactions using a three-track neural network. Science. 2021; **373**(6557): 871-6.
17. Krieger E, Vriend G. New ways to boost molecular dynamics simulations. J Comput Chem. 2015; **36**(13): 996-1007.
18. Trott O, Olson AJ. AutoDock Vina: Improving the speed and accuracy of docking with a new scoring function, efficient optimization, and multithreading. Journal of Computational Chemistry. 2010; **31**(2): 455-61.
19. Laskowski RA, Swindells MB. LigPlot+: Multiple Ligand–Protein Interaction Diagrams for Drug Discovery. Journal of Chemical Information and Modeling. 2011; **51**(10): 2778-86.
20. de Lau W, Barker N, Low TY, Koo BK, Li VS, Teunissen H, et al. Lgr5 homologues associate with Wnt receptors and mediate R-spondin signalling. Nature. 2011; **476**(7360): 293-7.
21. Bingham NC, Verma-Kurvari S, Parada LF, Parker KL. Development of a steroidogenic factor 1/Cre transgenic mouse line. Genesis. 2006; **44**(9): 419-24.
22. Bankhead P, Loughrey MB, Fernandez JA, Dombrowski Y, McArt DG, Dunne PD, et al. QuPath: Open source software for digital pathology image analysis. Sci Rep. 2017; **7**(1): 16878.

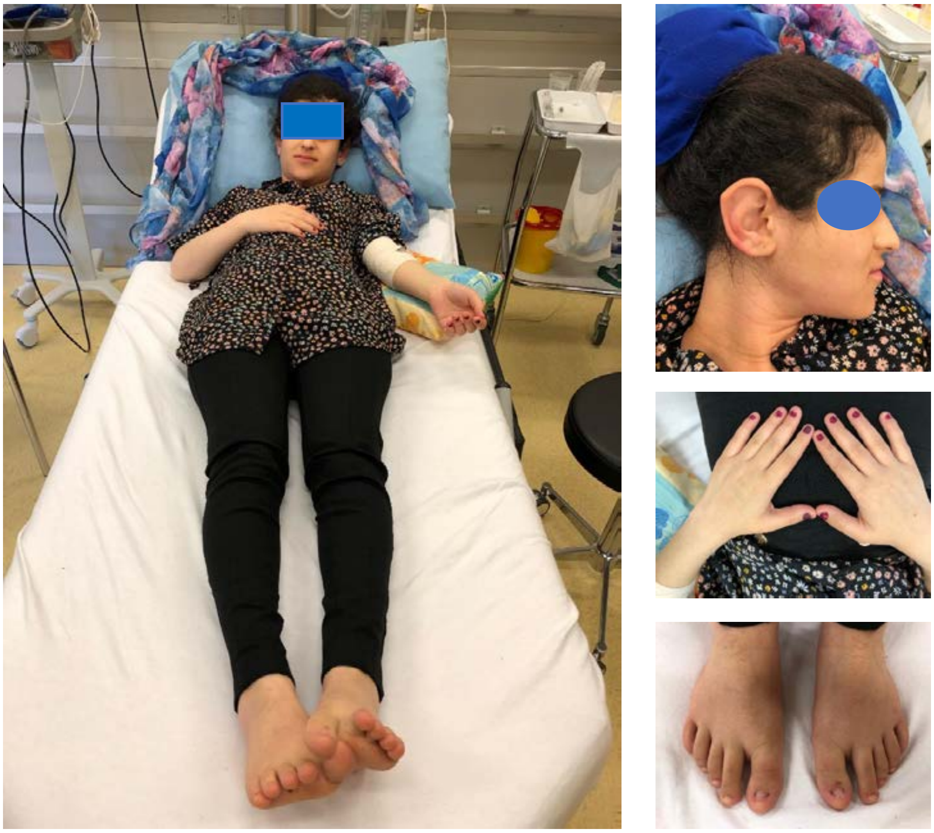

Supplementary Figure S1. Pictures of the 21-years-old index patient with a homozygote *LGR4* splice variant causing syndromic severe salt-wasting at birth. Note short stature (-3.1 SD), microcephaly (-3.5 SD) and dysplastic finger and toes nails. The young women is deaf and has some psychodevelopmental deficit. *Picture taken with consent of patient and both parents.*

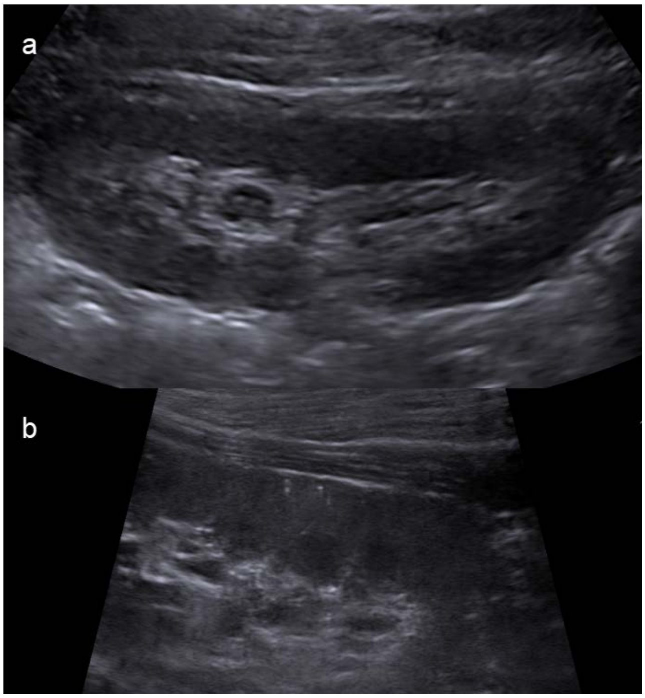

Supplementary Figure S2: Ultrasound of left and right kidneys showing diminished cortex to medulla demarcation, few triangular surface retractions and echo-rich cortical microlesions either corresponding to microcysts or microcalcifications *(Figure courtesy of Dr. Enno Stranzinger, Bern)*

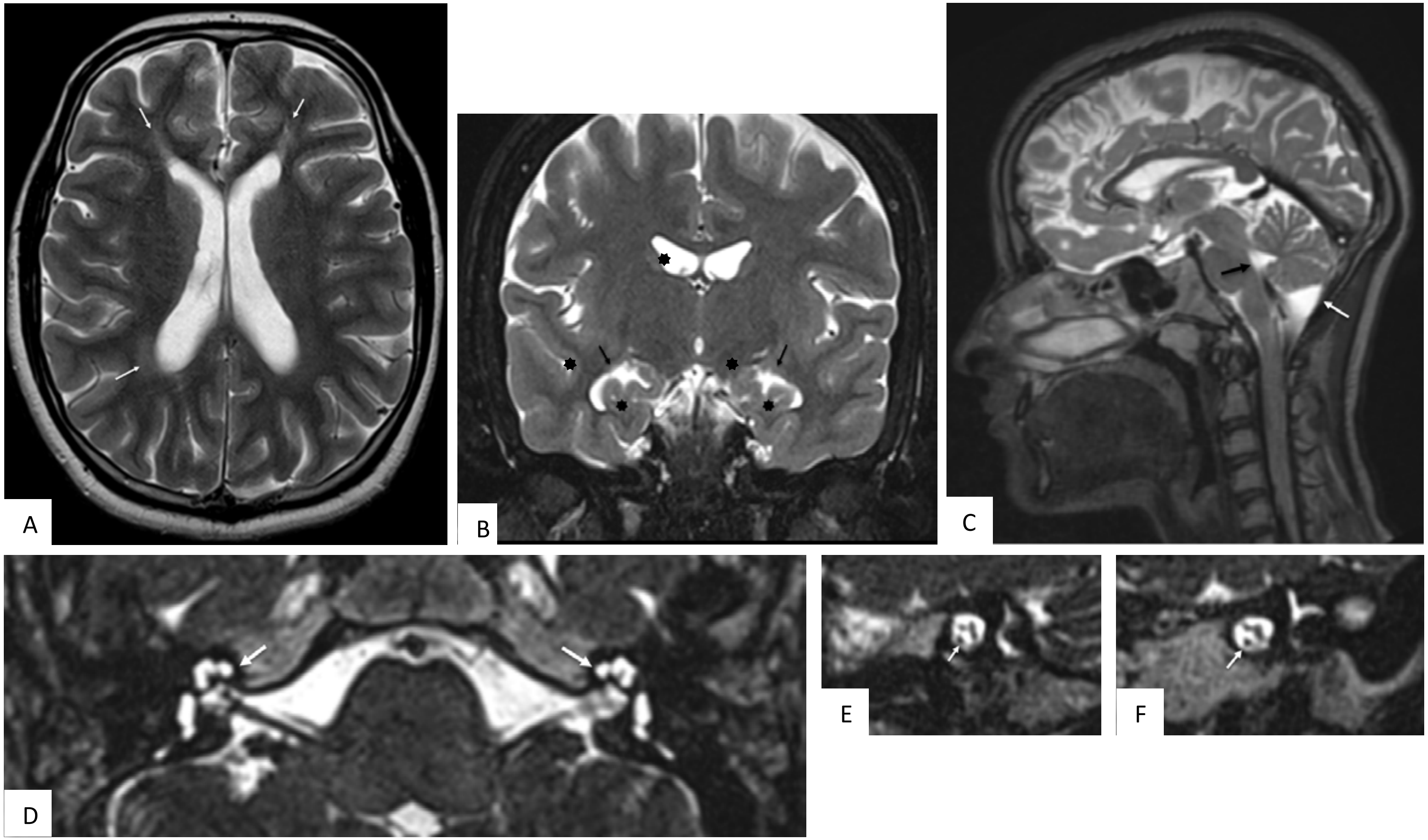

Supplementary Figure S3: Summary of MR imaging findings of the brain and inner ear structures. The patients was scanned on a our 1.5T Siemens Avanto MRI. Native and post contrast high resolutions sequences of the brain, inner ear and orbits were acquired. The axial T2-weighted image (A) shows periventricular hyperintensity (white arrows), indicating an incomplete myelination / incomplete cerebral maturation. That white matter hyperintensities are prominent seen around both anterior and the left occipital horns of the lateral ventricles. The fat-saturated coronal T2w (B) shows a noticeable malrotation of both hippocampi (black stars) with preservation of the internal architecture of both hippocampi. Associated there is slightly difference in the volume of the hippocampi with consecutive asymmetry and slightly enlargement of the choroidal fissure on both sides (B, black arrows). The posterior fossa is very small (C, white arrow). The sagittal T2w image revealed a flattening of the brainstem, especially at the pontomedullary junction (C, black arrow). The inner ear structures, especially the cochlea were inconspicuous on both sides in the axial CISS sequence (D, white arrows). The cochlear nerve appeared with normal caliber and regular course on both sides (E and F). The white arrows in E and F point to the right and left cochlear nerve in the parasagittal reconstructions of the CISS.

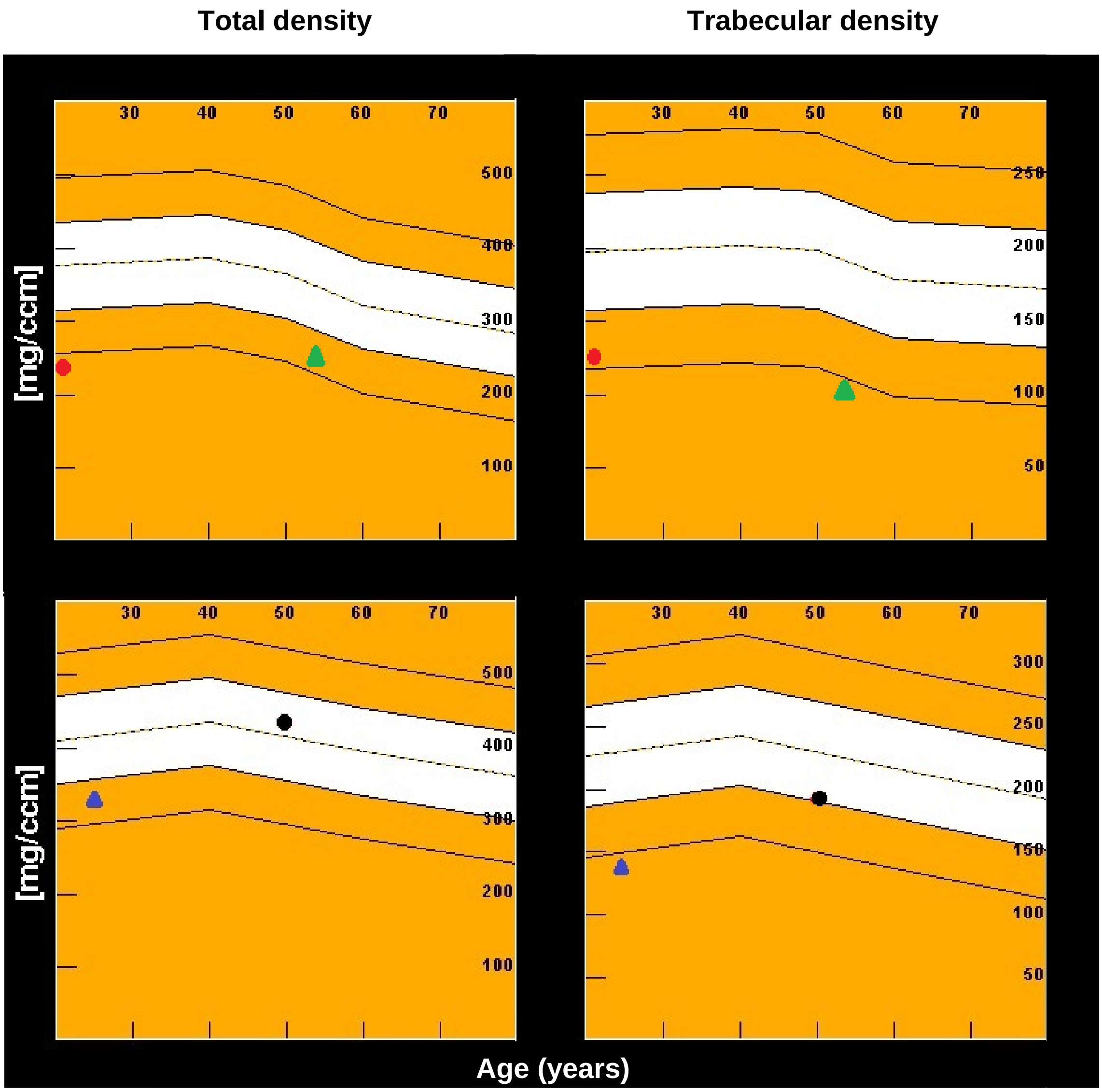

Supplementary Figure S4: Bone morphometric measurements of the index patient and family members. Measurements were performed with a Stratec XCT 3000 scanner (Stratec Medizintechnik, Pforzheim, Germany). PQCT measurements of the radius were performed on the non-dominant side and according to manufacturer's recommendations at 4% and 66% of the bone's total length measured from the reference line. Slice thickness was 2.2 mm, and voxel size was set at 0.5 mm with a scanning speed of 20 mm/s. Analysis were performed by manufacturer's software XCT 6.00 B (Stratec Medizintechnik, Pforzheim, Germany). Densitiy is shown as total density (on the left) and trabecular density (on the right) of females on the upper panel (homozygote index patient, red dot; heterozygote mother of index, green triangle) and of heterozygote males on the lower panel (brother, blue triangle; father, black dot). The white area indicates +/- 1 SD of the mean density in normal controls, the yellow area to the adjacent line +/- 2SD.

A

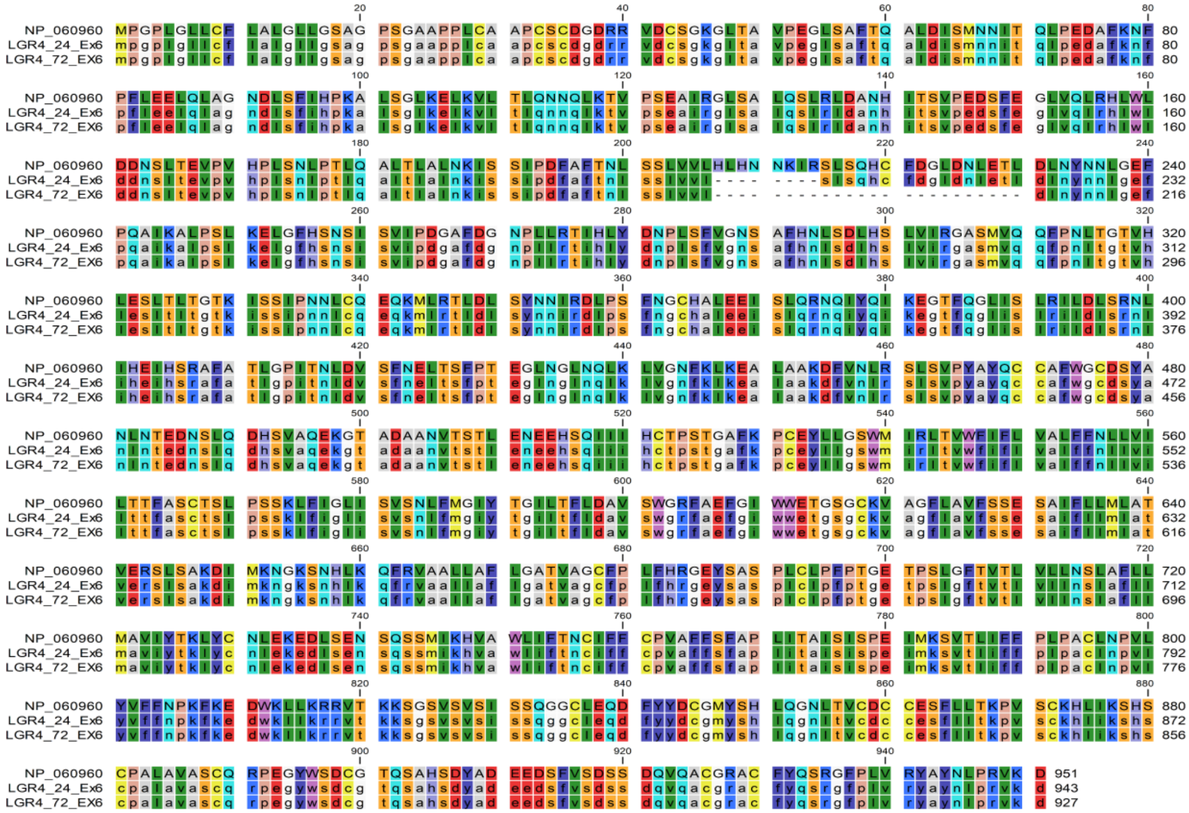

B

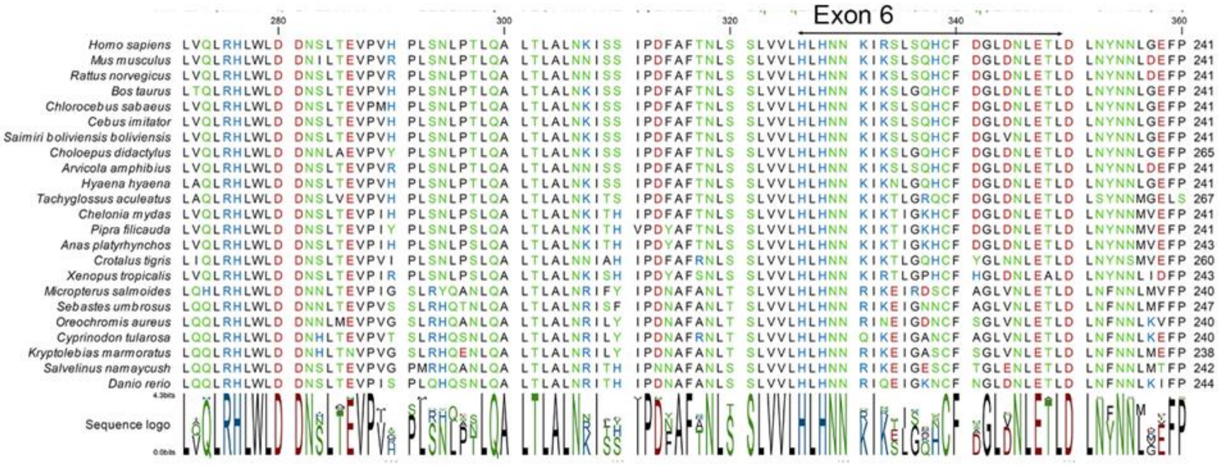

Supplementary Figure S5: A**.** Amino acid composition of WT (NP_060960) and mutant LGR4 proteins used in this study. B. Alignment of human RSPO4 with RSPO proteins across species. Only the region around Exon 6 is shown to highlight the conservation of amino acids involved in binding with RSPO proteins that are missing in the mutant version of LGR4 found in patients.

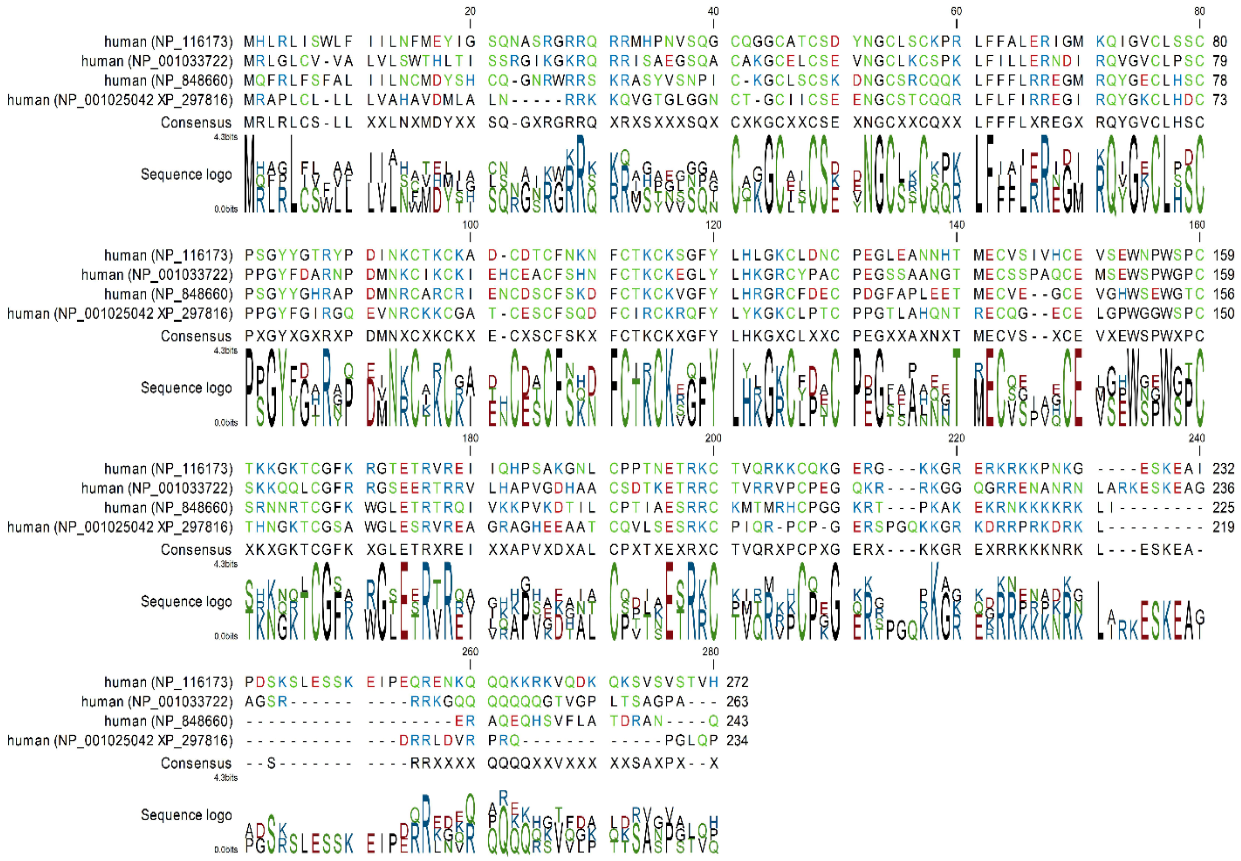

Figure S6: Amino acid sequence alignment of different human RSPO proteins. Sequences of RSPO1-4 were obtained from NCBI database and aligned with CLUSTALW. Amino acids involved in binding to LGR4 are highly conserved and RSPO3 (NP_116173) shares high sequence homology with the RSPO1 (NP_001033722). The high sequence similarity allowed us to analyze the binding of RSPO3 to LGR4 using the crystal structure of LGR4 bound to RSPO1 protein.

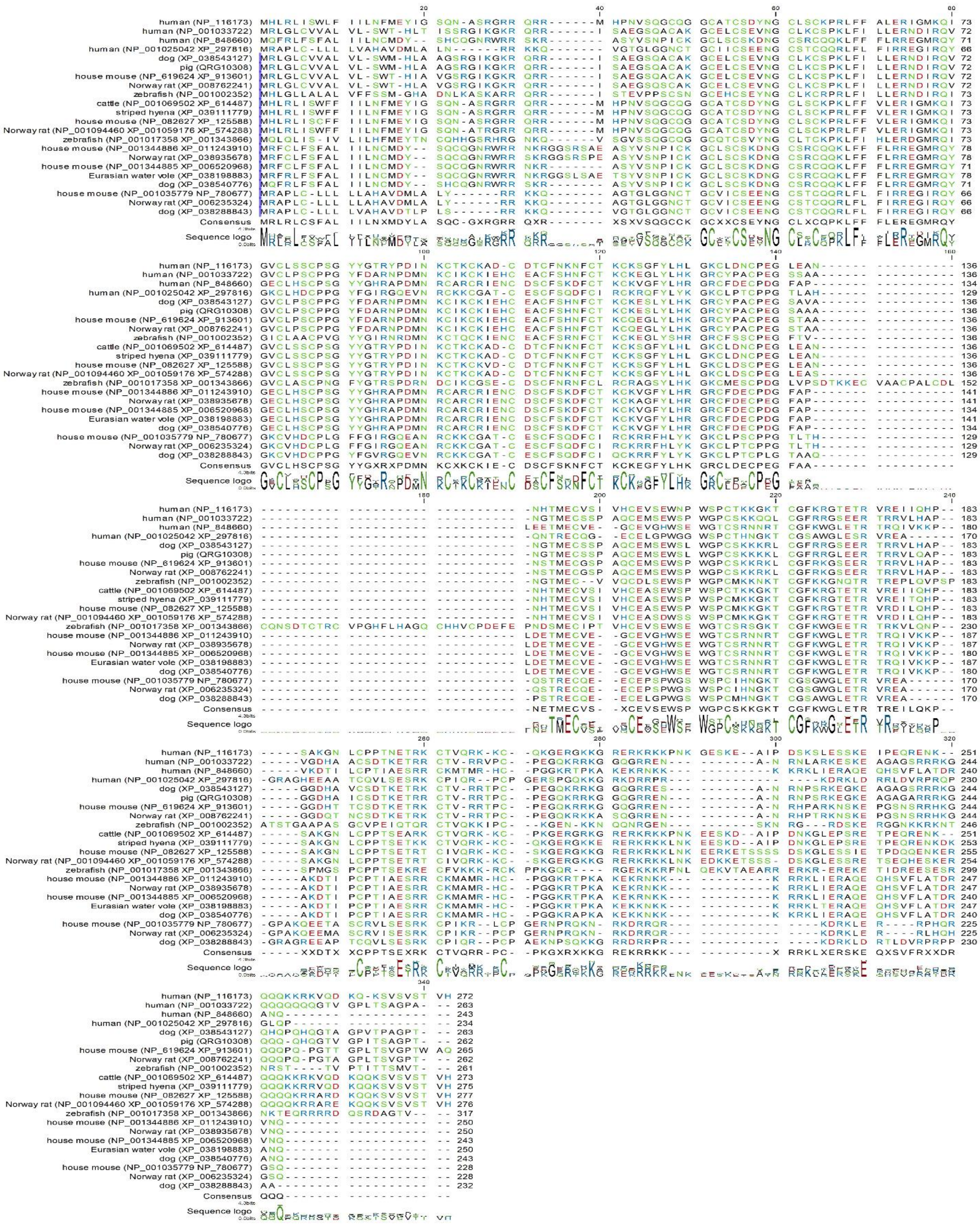

Figure S7: A multiple sequence alignment of RSPO1-4 proteins from human along with RSPO proteins from multiple other species to study the conservation of amino acids interacting with LGR4. Amino acids of RSPO proteins, like Arg 60 and Arg 88 are found in every single sequence analyzed.

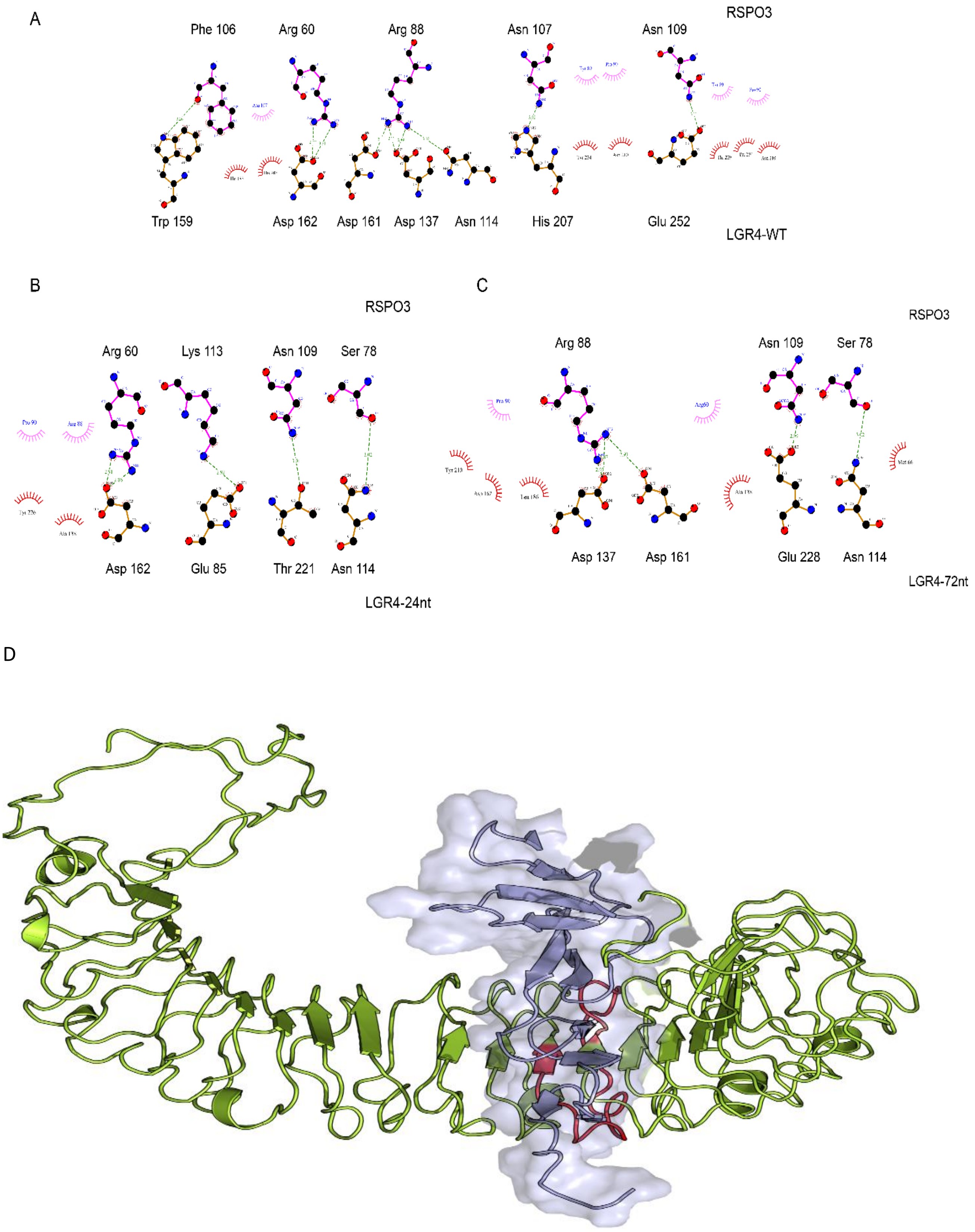
A
RSPO3
Phe 106
Arg 60
Arg 88
Asn 107
Asn 109
Trp 159
Asp 162
Asp 161
Asp 137
Asn 114
His 207
Glu 252
LGR4-WT
B
RSPO3
Arg 60
Lys 113
Asn 109
Ser 78
Asp 162
Glu 85
Thr 221
Asn 114
LGR4-24nt
C
RSPO3
Arg 88
Asn 109
Ser 78
Asp 137
Asp 161
Glu 228
Asn 114
LGR4-72nt
D

Figure S8: Interaction of LGR4 with RSPO3. A. contact points of LGR4-WT with RSPO3. Several amino acids located in LRR7 and LRR8 of LGR4 (His 207, Val 204, Asn 210, Thr 229, Tyr 234, and Glu 252) affected by deletion of exon 6 in LGR4. B. Changes in LGR4-RSPO3 contact points due to deletion of 24 nucleotides (8 AA) in LGR4 of patient. Multiple contacts between the two proteins are lost, including the crucial interaction points with Arg 88 of RSPO3. C. Contact of LGR4-72nt (-24 AA, deletion of exon 6). Deletion of whole exon 6 in LGR4 results in altered interaction profile and loss of multiple contacts between the two proteins, resulting in highly unstable interaction compared to WT LGR4. D. A closeup of LGR4-RSPO3 complex. LGR4 is shown as a ribbons model in green, while RSPO3 is shown as a surface and ribbons model in blue. Amino acids coded by exon 6 of LGR4 are shown in red. The amino acids deleted by loss of exon 6 in the patient are located exactly at the LGR4-RSPO3 interaction site and their loss would result in destabilization of the complex and downstream signaling.

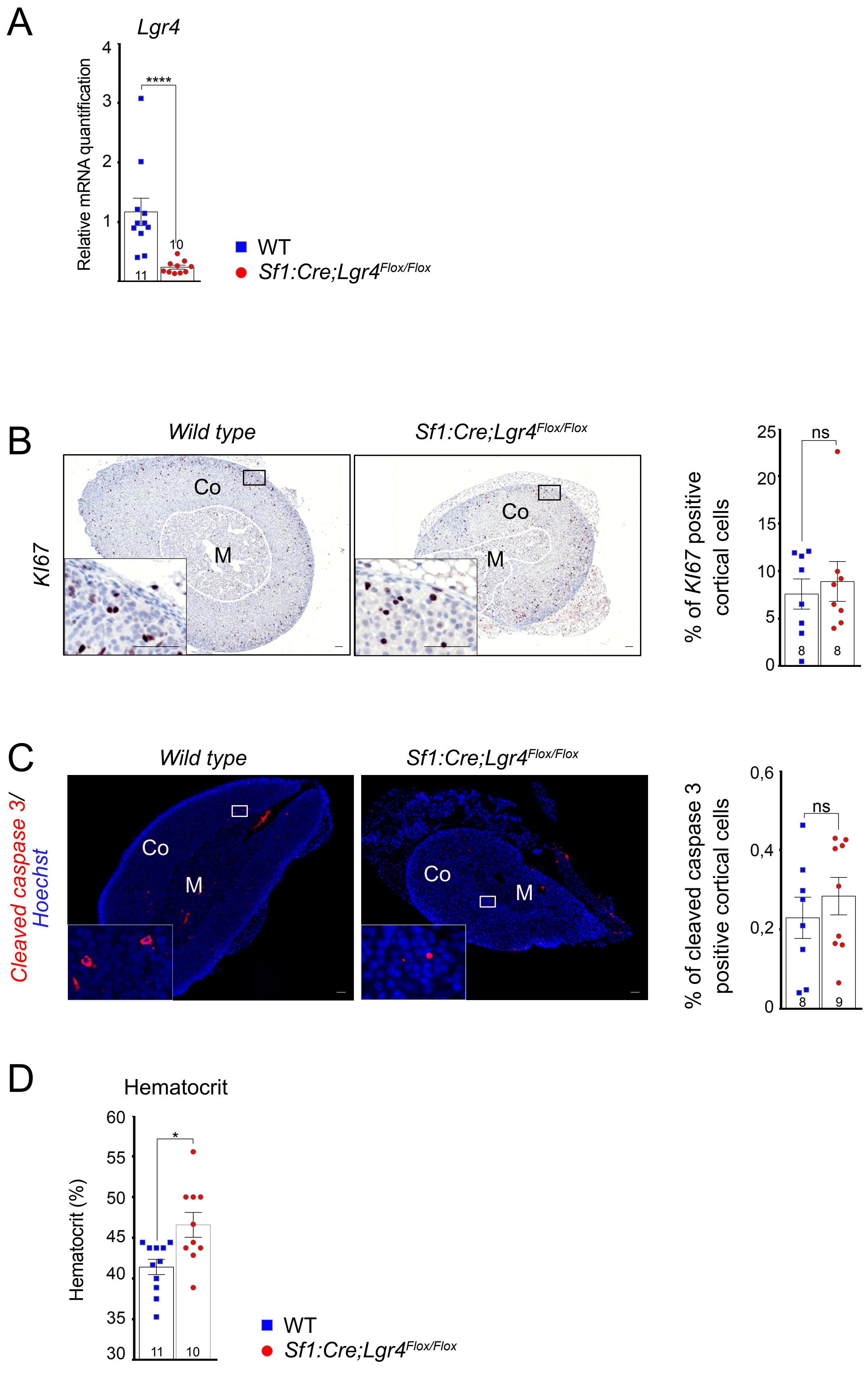

Supplementary Figure 9. A. RT-qPCR analysis of mRNA encoding *Lgr4* gene. **B.** Immunohistochemical detection and quantification of Ki67expression. **C.** Immunohistochemical detection and quantification of Cleaved Caspase 3. **D.** Quantification of hematocrit percentage. All analyses were conducted in 5 weeks wild type and Lgr4cKO female mice. Bars represent the mean expression ± SEM. Numbers of individual samples analysed are indicated within the bars. Statistical analyses in panels A, B, C & D were conducted using Mann-Whitney tests in GraphPad Prism 9. ns, not significant *P<0.05, ****P<0.0001.

Supplementary Table S1. Bone morphometrics in individuals carrying homozygous (index) and heterozygous *LGR4* variants.

A. Measurements were performed by GEHC-Lunar Prodigy (GEHC, Madison, WI, USA). BMD was measured at the lumbar spine (antero-posterior projection of L1 through L4) and the proximal femur (neck and trochanteric regions). Lean soft tissue mass was assessed for the entire body using GEHC-Lunar Prodigy. The measurements were performed in the array mode with standard positioning techniques. BMD and Body composition were analyzed using the DXA enCORE software (version 2004 8.80 001). T-Scores and Z-Scores refer to ISCD official positions 2019 [1] .

B. Volumetric Bone mineral density measurements were performed with a Stratec XCT 3000 scanner (Stratec Medizintechnik, Pforzheim, Germany). PQCT measurements of the radius were performed on the non-dominant side and according to manufacturer's recommendations at 4% and 66% of the bone's total length measured from the reference line. Slice thickness was 2.2 mm, and voxel size was set at 0.5 mm with a scanning speed of 20 mm/s. For Z-Scores the manufacturer's software XCT 6.00 B (Stratec Medizintechnik, Pforzheim, Germany) *or age-dependent reference data for pQCT analyses** were used [2].

**A**	**Index (21 yrs.)**		**Brother (26 yrs.)**		**Mother (55 yrs.)**		**Father (51 yrs.)**	
	BMD (mg/cm^2)	Z-score	BMD (mg/cm^2)	Z-score	BMD (mg/cm^2)	Z-score / T-score	BMD (mg/cm^2)	Z-score / T-score
**Areal BMD**								
- total Hip left	657	-2.2SD	720	-2.6SD	796	-1.1SD/ -1.7SD	980	-0.9SD/ -0.8SD
- femoral neck	601	-2.6SD	755	-2.2SD	750	-1.1SD/ -1.9SD	826	-1.7SD/ -1.9SD
- spine (L1-4)	905	-1.3SD	934	-1.9SD	828	-2.2SD/ -2.9SD	1052	-1.9SD/ -1.4SD
- whole body	777	-1.7SD	1000	-1.3SD	907	-1.1SD/ -1.7SD	1169	-1.3SD/ -0.3SD
**Fractures**	none		none		none		none	

B	Index (21 yrs)			Brother (26 yrs)			Mother (55 yrs)			Father (51 yrs)		
**Radius**		Z-value*	Z-value**		Z-value*	Z-value**		Z-value*	Z-value**	BMD	Z-value*	Z-value**
trab BMD (mg/cm^3)	123.62	-1.8 SD	-2.0 SD	141.02	-2.2 SD	-2.0 SD	100.91	-2.2SD		191.98	-0.9SD	
cort BMD (mg/cm^3)	1026.28		-3.0 SD	1066.83		-1.8SD	1019.42			1112.59		
Total BMD (mg/cm^3)	237.28	-2.3 SD	-	328.99	-1.5 SD		336.75	-1.6SD		433.3	0.3SD	
Total CSA (mm^2)	107.75			193.5			151.00			171.25		
cort CSA (mm^2)	42.75			69.50			45.75			93.5		

Table S2. List of alternative variants segregating with the clinical phenotype and an autosomal recessive mode of inheritance

Chr	Start	End	Ref	Alt	Gene	Transcript	cDNA	AAChange	OMIM	Function	Impact	GnomAD	CADD_PHRED	SIFT	PolyPhen Hum Var	dbSNP ID	ACMG classification	Adrenal expression*
chr7	881651	881651	C	T	SUN1	NM_001130965.3	c.335C>T	p.Thr112Met	* 607723	missense_variant	MODERATE	0.0003	10.69	Tolerated	Benign	rs183872262	VUS	33.67
chr10	88446830	88446830	G	A	LDB3	NM_001171610.1	c.694G>A	p.Asp232Asn	* 605906	missense_variant	MODERATE	0.0070	23.4	Tolerated	Benign	rs121908338	likely benign	0.46
chr10	93756290	93756290	C	A	BTAF1	NM_003972.3	c.3474C>A	p.Asp1158Glu	* 605191	missense_variant	MODERATE	no match	19.14	Tolerated	Benign	no match	VUS	18.91
chr10	102242476	102242476	G	T	WNT8B	NM_003393.4	c.959G>T	p.Arg320Leu	* 601396	missense_variant	MODERATE	3.489e-05	26.1	Damaging	Benign	rs773989054	VUS	0.083
**chr11**	**27405955**	**27405955**	**C**	**G**	**LGR4**	**NM_018490.5**	**c.618-1G>C**	**.**	*** 606666**	**splice_acceptor & intron_variant**	**HIGH**	**no match**	**na**	**na**	**na**	**no match**	**pathogenic**	**21.25**
chr15	86123809	86123809	C	T	AKAP13	NM_007200.5	c.2510C>T	p.Thr837Met	* 604686	missense_variant	MODERATE	0.0047	15.83	Tolerated	Benign	rs114703106	likely benign	11.1
chr15	86273745	86273745	A	C	AKAP13	NM_007200.5	c.7089A>C	p.Glu2363Asp	* 604686	missense & splice_region_variant	MODERATE	0.0025	45.55	Tolerated	Benign	rs116261620	likely benign	11.1
chr17	76823418	76823418	G	T	USP36	NM_001321291.2	c.598C>A	p.His200Asn	*612543	missense_variant	MODERATE	0.0047	22.8	Damaging	Benign	rs150547254	likely benign	15.29
chr19	53854857	53854857	G	A	ZNF845	NM_138374.3	c.929G>A	p.Arg310Gln	no match	missense_variant	MODERATE	0.0001	0.05	Tolerated	Benign	rs565297561	VUS	2.45

Filtering strategy: Variants kept were homozygous in the proband, heterozygous or absent in the parents, as well as heterozygous or absent in the unaffected siblings. Variants were kept if a low AF was found in control cohorts (<0.01 in GnomAD). Only variants annotated as missense variant, splicing region variant and nonsense variant with moderate or high impact were kept.

Abbr.: VUS - variant of uncertain significance; na - not applicable

*Median gene and transcript expression in the adrenals as of the GTEx Portal, numbers show Transcripts Per Million (TPM)

Supplementary Table S3. Phenotype of *LGR4* variants in humans and KO mice

	**Index family**			**Pubertal delay cohort** [1]	**Low bone density cohort** [2]	***Lgr4* mouse models**
**Genetic characteristics**	NM_018490.3 c.618-1G>C	NM_018490.3 c.618-1G>C		c.286A>G, c.1087G>T, c.2531A>G	c.376C>T	
	homozygote	het	*Phenocopies*	het	het	
	splice variant	splice variant		missense	nonsense	
	**Index**	**First degree relatives**	**Consanguineous cousins**	17 subjects of 6 families	Islandic GWAS cohort	
**Organ system**						
Fetal death	(in 3 siblings)	no	unknown			Yes [3,4]
Size at birth	normal	normal	normal		smaller	Yes [3,5]
Adult height	short stature	shorter	short stature	shorter	normal	
Adult weight/BMI	low	variable	low		lower	
Electrolyte imbalance	yes	no	yes		yes	Yes [6]
Aldosterone (MC)	decreased		low		normal	Increased [6]
Cortisol/corticosterone (GC)	subnormal		low			Normal [6]
Puberty/fertility:		variable pubertal delay		**pubertal delay**	pubertal delay	Pubertal delay Lgr4+/- [1]
.male		normal			lower testosterone	Structural and functional abnormalities (epididymides, testes) [4,7-10]
.female	pubertal delay	pubertal delay	pubertal delay			Lack of pubertal development and small gonads; abnormal sexual development (Wolffian ducts and somatic cells) [1,9-11]
Kidney	abnormal	unknown	unknown	normal	unspecific defects	Reduced size [3,9,12-15]
.Renin	increased		unknown			Increased [12]
Liver/gut	normal	normal by history	unknown			Reduced size [3,12,13]

Blood						High Hb and Hct [6,16]
Bone density	decreased	decreased	unknown		**reduced**	Reduced [5,17]
Hearing (hair cells, cochlea)	deafness	normal	deafness			Defective cochlea development [18]
Eye anomalies	vision problems	none	undefined vision problems	none	none	Variable [9,19,20]
Neurodevelopmen t/Brain	impaired/structural anomalies	normal	impaired	normal	normal	Neuroendocrine anomalies (GnRH network) [1,9]
Hair/nails/skin	dysplastic nails	normal	dysplastic nails			Hair follicle impairment [13,21,22]

References:

1. Mancini A, Howard SR, Marelli F, et al. LGR4 deficiency results in delayed puberty through impaired Wnt/beta-catenin signaling. JCI Insight 2020;5.
2. Styrkarsdottir U, Thorleifsson G, Sulem P, et al. Nonsense mutation in the LGR4 gene is associated with several human diseases and other traits. Nature 2013;497:517-20.
3. Mazerbourg S, Bouley DM, Sudo S, et al. Leucine-rich repeat-containing, G protein-coupled receptor 4 null mice exhibit intrauterine growth retardation associated with embryonic and perinatal lethality. Molecular endocrinology 2004;18:2241-54.
4. Mendive F, Laurent P, Van Schoore G, Skarnes W, Pochet R, Vassart G. Defective postnatal development of the male reproductive tract in LGR4 knockout mice. Dev Biol 2006;290:421-34.
5. Luo J, Yang Z, Ma Y, et al. LGR4 is a receptor for RANKL and negatively regulates osteoclast differentiation and bone resorption. Nat Med 2016;22:539-46.
6. Wang J, Li X, Ke Y, et al. GPR48 increases mineralocorticoid receptor gene expression. J Am Soc Nephrol 2012;23:281-93.
7. Hoshii T, Takeo T, Nakagata N, Takeya M, Araki K, Yamamura K. LGR4 regulates the postnatal development and integrity of male reproductive tracts in mice. Biol Reprod 2007;76:303-13.
8. Hsu PJ, Wu FJ, Kudo M, Hsiao CL, Hsueh AJ, Luo CW. A naturally occurring Lgr4 splice variant encodes a soluble antagonist useful for demonstrating the gonadal roles of Lgr4 in mammals. PLoS One 2014;9:e106804.
9. Yi T, Weng J, Siwko S, Luo J, Li D, Liu M. LGR4/GPR48 inactivation leads to aniridia-genitourinary anomalies-mental retardation syndrome defects. The Journal of biological chemistry 2014;289:8767-80.
10. Li XY, Lu Y, Sun HY, et al. G protein-coupled receptor 48 upregulates estrogen receptor alpha expression via cAMP/PKA signaling in the male reproductive tract. Development 2010;137:151-7.
11. Koizumi M, Oyama K, Yamakami Y, et al. Lgr4 controls specialization of female gonads in mice. Biol Reprod 2015;93:90.
12. Kato S, Matsubara M, Matsuo T, et al. Leucine-rich repeat-containing G protein-coupled receptor-4 (LGR4, Gpr48) is essential for renal development in mice. Nephron Exp Nephrol 2006;104:e63-75.
13. Kinzel B, Pikiolek M, Orsini V, et al. Functional roles of Lgr4 and Lgr5 in embryonic gut, kidney and skin development in mice. Dev Biol 2014;390:181-90.
14. Dang Y, Liu B, Xu P, et al. Gpr48 deficiency induces polycystic kidney lesions and renal fibrosis in mice by activating Wnt signal pathway. PLoS One 2014;9:e89835.

15. Mohri Y, Oyama K, Akamatsu A, Kato S, Nishimori K. Lgr4-deficient mice showed premature differentiation of ureteric bud with reduced expression of Wnt effector Lef1 and Gata3. Dev Dyn 2011;240:1626-34.
16. Song H, Luo J, Luo W, et al. Inactivation of G-protein-coupled receptor 48 (Gpr48/Lgr4) impairs definitive erythropoiesis at midgestation through down-regulation of the ATF4 signaling pathway. The Journal of biological chemistry 2008;283:36687-97.
17. Sun P, Jia K, Zheng C, et al. Loss of Lgr4 inhibits differentiation, migration and apoptosis, and promotes proliferation in bone mesenchymal stem cells. J Cell Physiol 2019;234:10855-67.
18. Zak M, van Oort T, Hendriksen FG, Garcia MI, Vassart G, Grolman W. LGR4 and LGR5 Regulate Hair Cell Differentiation in the Sensory Epithelium of the Developing Mouse Cochlea. Front Cell Neurosci 2016;10:186.
19. Kato S, Mohri Y, Matsuo T, et al. Eye-open at birth phenotype with reduced keratinocyte motility in LGR4 null mice. FEBS Lett 2007;581:4685-90.
20. Weng J, Luo J, Cheng X, et al. Deletion of G protein-coupled receptor 48 leads to ocular anterior segment dysgenesis (ASD) through down-regulation of Pitx2. Proceedings of the National Academy of Sciences of the United States of America 2008;105:6081-6.
21. Ren X, Xia W, Xu P, et al. Lgr4 Deletion Delays the Hair Cycle and Inhibits the Activation of Hair Follicle Stem Cells. J Invest Dermatol 2020;140:1706-12 e4.
22. Mohri Y, Kato S, Umezawa A, Okuyama R, Nishimori K. Impaired hair placode formation with reduced expression of hair follicle-related genes in mice lacking Lgr4. Dev Dyn 2008;237:2235-42.

Antibody	Species	Dilution factor	Source	Catalog number
AKR1B7	Rabbit	1:600	Homemade	
Beta catenin	Mouse	1:500	BD Biosciences	610153
Cleaved caspase 3	Rabbit	1:200	Cell signaling	9661
CYP11B2	Rabbit	1:500	Gift, Gomez Sanchez	
DAB2	Mouse	1:500	BD Biosciences	610464
KI67	Rabbit	1:1500	Abcam	ab15580
LEF1	Rabbit	1:200	Abcam	ab137872

Supplemental table S4 : List of antibodies used for immunohistochemistry. Akr1b7 : aldo-keto reductase family 1, member B7 ; Cyp11b2 : cytochrome P450 family 11 subfamily B member 2 ; Dab2 : disabled homolog 2 ; Lef1 : lymphoid enhancer binding factor 1.

Gene symbol	Forward Primer	Reverse Primer
Akr1b7	GCCAGTGACCAACCAGATTGAGA	ACGGGGTCTTCTGGCTTGGCAT
Apcdd1	CTCAGCCCCACACTCATTCC	TGGCACGGAGTTTGTGTTCA
Axin2	ATGGGGAGTAAGAAACAGCTCC	CCAGCTCCAGTTTCAGTTTCTC
Beta catenin	AGTGCAGGAGGCCGAGG	GAGTAGCCATTGTCCACGCA
Cyp11a1	TACTAACCTAGCCCGCCTCG	GCTCCTGCGCATAGAGAGAG
Cyp11b1	GCAGAGATGATGCTCCTGCTT	GAGAGGGCAATGTGTCATCAGAA
Cyp21	GCTGTGGCTTTCCTGCTTCAC	GGCCCAGCTTGAGGTCTAACT
Dab2	CCTGCATCTTCTGATCCCCAC	CATGTTTCTGGCTGTCTGCTT
Hsd3b1	CCTACATTCTGAACTGAGCGGCTGC	GGTCTGTCCTTCCCAGTGATTGATAAAC
Hsd3b6	CATCCTTCCACAGTTCTAGC	TGGTGTGAGATTAATGTACA
Lef1	GACGAGCACTTTTCTCCGGG	TGGGGTGATCTGTCCAACGC
Lgr4	GCTGCGGACTCTGGACTTAT	TCGCAAAAGCTCCACTGTGA
Star	TCGCTACGTTCAAGCTGTGT	ACGTCGAACTTGACCCATCC

Supplemental table S5 : List of primers used for quantitative real-time PCR analysis. Akr1b7 : aldo-keto reductase family 1, member B7 ; Apcdd1 : adenomatosis polyposis down-regulated 1 ; Axin2 : axis inhibition protein 2 ; Cyp11a1 : cytochrome P450 family 11 subfamily A member 1 ; Cyp11b1 : cytochrome P450 family 11 subfamily B member 1 ; Cyp21 : cytochrome P450 Family 21 ; Dab2 : disabled homolog 2 ; Hsd3b1 : hydroxy-delta-5-steroid dehydrogenase, 3 beta- and steroid delta-isomerase 1 ; Hsd3b6 : hydroxy-delta-5-steroid dehydrogenase, 3 beta- and steroid delta-isomerase 6 ; Lef1 : lymphoid enhancer binding factor 1 ; Lgr : leucine rich repeat containing G protein-coupled receptor ; Star : steroidogenic acute regulatory protein.
